# Evaluation of FGFR targeting in breast cancer through interrogation of patient-derived models

**DOI:** 10.1186/s13058-021-01461-4

**Published:** 2021-08-03

**Authors:** Nicole J. Chew, Terry C. C. Lim Kam Sian, Elizabeth V. Nguyen, Sung-Young Shin, Jessica Yang, Mun N. Hui, Niantao Deng, Catriona A. McLean, Alana L. Welm, Elgene Lim, Peter Gregory, Tim Nottle, Tali Lang, Melissa Vereker, Gary Richardson, Genevieve Kerr, Diana Micati, Thierry Jardé, Helen E. Abud, Rachel S. Lee, Alex Swarbrick, Roger J. Daly

**Affiliations:** 1 Cancer Program, Monash Biomedicine Discovery Institute, Clayton, VIC 3800 Australia; 2 Department of Biochemistry and Molecular Biology, Monash University, Melbourne, VIC 3800 Australia; 3 Garvan Institute of Medical Research, Darlinghurst, NSW 2010 Australia; 4 St Vincent’s Clinical School, Faculty of Medicine, UNSW Sydney, Darlinghurst, NSW 2010 Australia; 5 Anatomical Pathology, Alfred Hospital, Prahran, VIC 3004 Australia; 6 Huntsman Cancer Institute, Salt Lake City, UT 84112 USA; 7 St Vincent’s Hospital, Darlinghurst, NSW 2010 Australia; 8 Cabrini Health, Brighton, VIC 3186 Australia; 9 TissuPath, Mount Waverley, VIC 3149 Australia; 10 Szalmuk Family Department of Medical Oncology, Cabrini Institute, Malvern, VIC 3144 Australia; 11 Development and Stem Cells Program, Monash Biomedicine Discovery Institute, Clayton, VIC 3800 Australia; 12 Department of Anatomy and Developmental Biology, Monash University, Clayton, VIC 3800 Australia

**Keywords:** Targeted therapy, Oncogene, Fibroblast growth factor receptor, SKI proto-oncogene, Precision oncology

## Abstract

**Background:**

Particular breast cancer subtypes pose a clinical challenge due to limited targeted therapeutic options and/or poor responses to the existing targeted therapies. While cell lines provide useful pre-clinical models, patient-derived xenografts (PDX) and organoids (PDO) provide significant advantages, including maintenance of genetic and phenotypic heterogeneity, 3D architecture and for PDX, tumor–stroma interactions. In this study, we applied an integrated multi-omic approach across panels of breast cancer PDXs and PDOs in order to identify candidate therapeutic targets, with a major focus on specific FGFRs.

**Methods:**

MS-based phosphoproteomics, RNAseq, WES and Western blotting were used to characterize aberrantly activated protein kinases and effects of specific FGFR inhibitors. PDX and PDO were treated with the selective tyrosine kinase inhibitors AZD4547 (FGFR1-3) and BLU9931 (FGFR4). FGFR4 expression in cancer tissue samples and PDOs was assessed by immunohistochemistry. METABRIC and TCGA datasets were interrogated to identify specific FGFR alterations and their association with breast cancer subtype and patient survival.

**Results:**

Phosphoproteomic profiling across 18 triple-negative breast cancers (TNBC) and 1 luminal B PDX revealed considerable heterogeneity in kinase activation, but 1/3 of PDX exhibited enhanced phosphorylation of FGFR1, FGFR2 or FGFR4. One TNBC PDX with high FGFR2 activation was exquisitely sensitive to AZD4547. Integrated ‘omic analysis revealed a novel FGFR2-SKI fusion that comprised the majority of FGFR2 joined to the C-terminal region of SKI containing the coiled-coil domains. High FGFR4 phosphorylation characterized a luminal B PDX model and treatment with BLU9931 significantly decreased tumor growth. Phosphoproteomic and transcriptomic analyses confirmed on-target action of the two anti-FGFR drugs and also revealed novel effects on the spliceosome, metabolism and extracellular matrix (AZD4547) and RIG-I-like and NOD-like receptor signaling (BLU9931). Interrogation of public datasets revealed FGFR2 amplification, fusion or mutation in TNBC and other breast cancer subtypes, while FGFR4 overexpression and amplification occurred in all breast cancer subtypes and were associated with poor prognosis. Characterization of a PDO panel identified a luminal A PDO with high FGFR4 expression that was sensitive to BLU9931 treatment, further highlighting FGFR4 as a potential therapeutic target.

**Conclusions:**

This work highlights how patient-derived models of human breast cancer provide powerful platforms for therapeutic target identification and analysis of drug action, and also the potential of specific FGFRs, including FGFR4, as targets for precision treatment.

**Supplementary Information:**

The online version contains supplementary material available at 10.1186/s13058-021-01461-4.

## Background

Breast cancer is the most commonly diagnosed cancer in women, with an estimate of more than 2 million new cases and more than 600,000 deaths in 2018 [1]. Global gene expression profiling has distinguished at least four intrinsic breast subtypes that include luminal A, luminal B, HER2 enriched and basal-like [2]. The basal-like subtype substantially overlaps with the triple-negative breast cancer (TNBC) subgroup (~ 80% of TNBC are basal-like) [3]. TNBC is the most aggressive subtype, associated with higher metastasis rate and tumor grade [4, 5] and lacks the expression of estrogen receptor (ER), progesterone receptor (PR) and HER2, ruling out endocrine and trastuzumab therapies as treatment options [5]. While chemotherapy remains the ‘backbone’ of TNBC treatment, recent developments include the use of PARP inhibitors for BRCA mutant TNBC and targeting the PD1 axis via immunotherapy [6]. However, given the paucity of effective targeted treatments for this disease subtype, this remains an intense area of investigation. In addition, the luminal B subtype is characterized by increased proliferation compared to luminal A cancers, relative resistance to chemotherapy, and a relatively poor outcome with endocrine therapy considering its ER-positive status [7].

Aberrant activation of specific receptor tyrosine kinases (RTKs) commonly occurs in human cancer, leading to the development of targeted approaches, including small molecule drugs, to block their activity [8]. Fibroblast growth factor receptors 1–4 (FGFR1-4) form a family of four highly conserved RTKs and deregulation of FGFR signaling, reflecting gene mutation, translocation, amplification and/or overexpression, occurs in a variety of human malignancies including urothelial (32% of cases) and breast cancers (18%) [9, 10]. Successful pre-clinical demonstration of the efficacy of FGFR targeting, for example, using selective small molecule drugs, has led to evaluation of such approaches in human clinical trials. For example, in a translational clinical trial, 12.5 and 33% of gastric cancers exhibiting FGFR1- and FGFR2-amplification, respectively, exhibited responses to the FGFR1-3 inhibitor AZD4547 [11]. Furthermore, the pan-FGFR kinase inhibitor BGJ398 (Infigratinib) demonstrated significant activity against chemotherapy-refractory cholangiocarcinoma harboring FGFR2 fusions in a phase II clinical study (NCT02150967) [12]. Recently, Erdafitinib (an inhibitor of FGFR1-4) was FDA-approved for patients with metastatic urothelial carcinoma exhibiting FGFR gene alterations and resistance to chemotherapy, based on a phase II clinical trial results [13]. Erdafitinib, as well as other selective FGFR inhibitors including Infigratinib, Pemigatinib and Rogaratinib, are currently being evaluated in late-stage clinical trials in several solid malignancies [14]. While the initial focus of FGFR targeting was FGFR1-3, FGFR4 has recently attracted significant interest. The FGFR4 ligand FGF19 is often overexpressed in hepatocellular carcinoma (HCC) due to focal amplification of chromosome 11q13.3 [15, 16]. Selective FGFR4 inhibitors are currently in early stage clinical trials for treatment of HCC (NCT02834780) [14].

In breast cancer, FGFR1 amplification occurs in 14% of cases, and FGFR1 expression is an independent negative prognostic factor in TNBC [17, 18]. FGFR1 amplification is also associated with poor prognosis in ER-positive cancers and confers resistance to endocrine therapies [19, 20]. Similarly, FGFR2 is also positively associated with poor prognosis and endocrine resistance [21–23]. Increased FGFR3 expression is significantly associated with reduced overall survival and FGFR3-TACC3 fusions have been detected in a primary TNBC and the TNBC cell line SUM-185PE, with an oncogenic driver role defined in the latter context [24, 25]. Finally, increasing evidence supports subtype-selective roles for FGFR4 in breast cancer. An activating mutation (Y367C) in this receptor leads to an oncogenic role in the TNBC cell line MDA-MB-453 [26] and a single nucleotide polymorphism (SNP) (Gly388Arg) is also associated with reduced overall survival in breast cancer patients receiving adjuvant systemic therapy [27]. In addition, FGFR4 is implicated in metastasis and endocrine resistance in invasive lobular carcinoma [28], and a recent study indicates that FGFR4 promotes transition from a more differentiated, luminal phenotype to a highly proliferative and metastatic, HER2-enriched one [29].

Previously, we integrated global phosphoproteomic profiling of human breast cancer cell lines and genetically modified mouse models of this disease with functional analyses in order to identify subtype-selective signaling networks and candidate therapeutic targets [24, 30, 31]. In this study, we have extended this approach to breast cancer patient-derived xenografts (PDX) and organoids (PDO), powerful models that retain the genetic and phenotypic heterogeneity of the primary tumor, exhibit 3D architecture and for PDX, tumor–stroma interactions [32–34]. Our findings, which include characterization of oncogene addiction to a novel FGFR2 fusion in a TNBC PDX and identification of an important role for FGFR4 in a subset of luminal breast cancers, support and widen opportunities for therapeutic targeting of specific FGFRs as a strategy for precision treatment of breast cancer.

## Materials and methods

### Cell lines, cell culture and reagents

CAL120 cells were a gift from Elgene Lim (Garvan Institute of Medical Research, Darlinghurst, NSW 2010, Australia). MFM-223 cells were purchased from Sigma-Aldrich. MDA-MB-453 cells were purchased from ATCC (Manassas, VA, USA). SUM185PE cells were purchased from Asterand Bioscience. Cells were cultured in RPMI-1640 (Gibco) and supplemented with 10% (v/v) FBS (Moregate), 10 μg/ml Actrapid penfill insulin (Clifford Hallam Healthcare) and 20 mM HEPES (Gibco).

For harvesting, cells at 80% confluency were washed twice with ice cold 1 × PBS then lysed with RIPA buffer (0.5% (w/v) sodium deoxycholate, 150 mM NaCl, 1% (v/v) NP40, 50 mM Tris–HCl pH 8.0, 0.1% (w/v) SDS, 10% (v/v) glycerol, 5 mM EDTA and 20 mM NaF), supplemented with 10 μg/ml aprotinin, 1 mM PMSF, 10 μg/ml leupeptin, 1 mM sodium orthovanadate, 2.5 mM sodium pyrophosphate and 2.5 mM β-glycerophosphate prior to use. Lysed cells were collected and clarified by centrifugation at 21,130 × *g* at 4 °C for 10 min, then the protein concentration was determined using a Pierce BCA protein assay kit (Thermo Scientific) according to the manufacturer’s protocol.

### Tyrosine phosphorylation profiling by mass spectrometry

To harvest protein lysates for mass spectrometry (MS) analysis, snap-frozen PDX samples were homogenized and lysed with lysis buffer (6 M guanidine hydrochloride, 50 mM Tris–HCl, 1 mM sodium orthovanadate, 2.5 mM sodium pyrophosphate, 1 mM b-glycerophosphate). Approximately 20 mg of lysate protein was reduced with 5 mM TCEP at 37 °C for 1 h and alkylated with iodoacetamide in the dark for 1 h. The samples were then diluted 1:4 with ammonium bicarbonate (25 mM) before digestion with 1:200 LysC (Worthington) at room temperature (RT) for 4 h. Samples were further diluted 10 × from the original volume before digestion with trypsin (Promega) (1:100) at 37 °C for 18 h. Tryptic digests were acidified with 10% TFA to pH 3 before desalting on a C18 column (Thermo Fisher Scientific) and elution with 0.1% TFA/40% ACN. Peptides were dried in a vacuum concentrator (CentriVap™ Labconco) and reconstituted in 1.8 ml of IAP wash buffer (1% n-octyl-b-d-glucopyranoside, 50 mM Tris–HCl, 150 mM NaCl, pH 7.4). 50 μg each of P-Tyr-1000 (Cell Signaling Technology, 8954), P-Tyr-100 (Cell Signaling Technology, 9411) and P-Tyr-20 (BD Biosciences, 610000) antibodies were coupled to 60 μL of sepharose beads slurry (Rec-Protein G, Zymed) and incubated overnight with peptide samples at 4 °C with gentle shaking. Immobilized antibody beads were washed three times with IAP buffer and further washed three times with water before elution with 110 μL of 0.15% TFA. Samples were then desalted on a C18 column (as described above) and evaporated to dryness in a vacuum concentrator. The dried peptides were reconstituted in MS loading buffer (2% ACN/0.5% FA).

### TiO_2_ enrichment for mass spectrometry analysis

PDX samples were resuspended and homogenized in 4% cold sodium deoxycholate (SDC) lysis buffer (4% w/v SDC in 100 mM Tris–HCl pH 8.5). To facilitate lysis and to inactivate endogenous proteases and phosphatases, the samples were then boiled at 95 °C for 5 min prior to centrifugation at 1500 g for 5 min. Protein concentration was measured by BCA and 200 ug of protein was reduced and alkylated with 10 mM TCEP and 40 mM 2-chloroacetamide (pH 8.5) for 5 min at 95 °C. Samples were allowed to cool to RT prior to digestion with Lys-C (1:200 (w/w)) and Trypsin (1:100 (w/w)) and incubated for ~ 16 h at 37 °C with constant shaking at 1500 rpm. The enzymatic reaction was stopped with the sequential addition of 400 ul isopropanol and 100 ul EP enrichment buffer (48% (v/v) TFA and 8 mM KH2PO4), with thorough mixing (1500 rpm for 30 s) after each addition. Samples were centrifuged at 2000 g for 15 min at RT and the cleared lysates were collected for the enrichment step. TiO_2_ beads were added to the lysates at a ratio of 12:1 (w/w) bead to protein ratio and incubated at 40 °C with shaking (2000 rpm) for 5 min. The TiO_2_ beads were pelleted by centrifugation (2000 g for 1 min at RT) and the supernatant was carefully aspirated. The beads were washed four times with 1 ml EP wash buffer (5% (v/v) TFA and 60% (v/v) Isopropanol). After the final wash, the beads were resuspended in 75 µl EP transfer buffer (0.1% TFA/60% (v/v) isopropanol) and transferred onto a C8 StageTip (Thermo Fisher Scientific). An additional 75 µl of EP wash buffer was used to capture any remaining beads. The C8 StageTips were centrifuged at 1500 g for ~ 8 min at RT, until all EP wash buffer had passed through. The phosphopeptides were eluted twice with 30 µl EP elution buffer (5% ammonia solution (NH4OH) in 40% (v/v) acetonitrile) and centrifuged to dryness at 1500 g for ~ 4 min at RT. The eluates were dried down to < 15 µl in a vacuum concentrator, resuspended in 100 µl SDS-RPS wash buffer 1 (1% (v/v) TFA in Isopropanol) and transferred onto SDB-RPS StageTips (CDS Empore™). The StageTips were centrifuged to dryness at 1500 g for ~ 8 min at RT, followed by sequential washes with 100 µl SDS-RPS wash buffer 1 and 100 µl SDS-RPS wash buffer 2 (0.2% (v/v) TFA and 5% (v/v) acetonitrile). The phosphopeptides were then eluted with 60 µl SDS-RPS elution buffer (0.125% NH4OH solution in 60% (v/v) acetonitrile) and eluates were completely dried down in a vacuum concentrator. Samples were reconstituted in MS loading buffer prior to analysis by mass spectrometry by data-dependent acquisition (DDA).

### Mass spectrometry analysis

Samples were analyzed on an UltiMate 3000 RSLC nano-LC system (Thermo Fisher Scientific) coupled to an LTQ-Orbitrap mass spectrometer (LTQ-Orbitrap, Thermo Fisher Scientific). Peptides were loaded via an Acclaim PepMap 100 trap column (100 μm × 2 cm, nanoViper, C18, 5 μm, 100 Å, Thermo Fisher Scientific) and subsequent peptide separation was on an Acclaim PepMap RSLC analytical column (75 μm × 50 cm, nanoViper, C18, 2 μm, 100 Å, Thermo Fisher Scientific). For each liquid chromatography–tandem mass spectrometry (LC–MS/MS) analysis, 1 µg of peptides as measured by a nanodrop 1000 spectrophotometer (Thermo Fisher Scientific) was loaded on the pre-column with microliter pickup. Peptides were eluted using a 2 h linear gradient of 80% ACN/0.1% FA at a flow rate of 250 nL/min using a mobile phase gradient of 2.5–42.5% ACN. The eluting peptides were interrogated with an Orbitrap mass spectrometer. The HRM DIA method consisted of a survey scan (MS1) at 35,000 resolution (automatic gain control target 5e6 and maximum injection time of 120 ms) from 400 to 1220 *m*/*z* followed by tandem MS/MS scans (MS2) through 19 overlapping DIA windows increasing from 30 to 222 Da. MS/MS scans were acquired at 35,000 resolution (automatic gain control target 3e6 and auto for injection time). Stepped collision energy was 22.5%, 25%, 27.5% and a 30 *m*/*z* isolation window. The spectra were recorded in profile type.

For the DDA acquisition, the LC method was the same as for the HRM-DIA described above. The following settings were applied to the mass spectrometer operated in DDA mode. Survey full scan MS spectra (*m*/*z* 375–1800) were acquired in the Orbitrap with 70,000 resolution (at *m*/*z* 200) after accumulation of ions to a 3 × 10^6^ target value with a maximum injection time of 30 ms. Dynamic exclusion was set to 20 s. The 10 most intense charged ions (z ≥  + 2) were sequentially isolated and fragmented in the collision cell by higher-energy collisional dissociation (HCD) with a fixed injection time of 60 ms, 30,000 resolution and AGC target of 5 × 10^4^.

### HRM-DIA data analysis

The DIA data were analyzed with Spectronaut 8, a mass spectrometer vendor-independent software from Biognosys. Default settings were applied for the Spectronaut search. Retention time prediction type was set to dynamic indexed retention time (iRT; correction factor for window 1). Decoy generation was set to scrambled (no decoy limit). Interference correction on MS2 level was enabled. The false discovery rate (FDR) was set to 1% at peptide level. A peptide identification required at least 3 transitions in quantification. Quantification was based on the top 3 proteotypic peptides for each protein, normalized with the default settings and exported as an excel file with Spectronaut 8 software [42]. For generation of the spectral libraries, DDA measurements of each sample were taken. The DDA spectra were analyzed with the MaxQuant Version 1.5.2.8 analysis software using default settings. Enzyme specificity was set to Trypsin/P, minimal peptide length of 6, and up to 3 missed cleavages were allowed. Search criteria included carbamidomethylation of cysteine as a fixed modification; oxidation of methionine; acetyl (protein N terminus); and phosphorylation of serine, threonine and tyrosine as variable modifications. The mass tolerance for the precursor was 4.5 ppm and for the fragment ions was 20 ppm. The DDA files were searched against a concatenated human (v2015-08, 20,210 entries) and mouse (v2015-10, 16,983 entries) UniProt fasta database and the Biognosys HRM calibration peptides. The identifications were filtered to satisfy FDR of 1% on peptide and protein level. The spectral library was generated in Spectronaut and normalized to iRT peptides.

### TiO_2_ enrichment DDA data analysis

DDA raw files were analyzed on MaxQuant using the same setting as for the spectral library generation described above. The only differences were enzymatic specificity was set to Trypsin/P and LysC/P, and the Uniport fasta databases were dated v2020-03 with 20,350 and 17,009 entries for human and mouse, respectively. Match between runs was selected using MaxQuant’s default settings. R packages were used for normalization (NormalyzerDE), imputation of missing values (impute.knn) and differential analysis (limma).

### Patient-derived xenograft propagation and tissue collection

PDX models were provided by the Brocade consortium (https://www.petermac.org/research/research-cohort-studies/brocade) and 2 models were previously published [35]. Viably frozen PDX tumor tissue was first propagated and expanded into 3 immunodeficient mice per PDX model. Briefly, 1 mm^3^ tumor pieces were implanted into the fourth mammary fat pad of NSG mice. Twice weekly standard monitoring and tumor measurement were conducted, and once tumors reached appropriate size, ~ 1000 mm^3^, mice were sacrificed by cervical dislocation under deep, isoflurane-induced anesthesia. Tumors were harvested and cryopreserved prior to passaging into mice for drug studies. Mice were enrolled for drug or vehicle control treatment when tumors reached 200 mm^3^ for the short-term studies, and 100 mm^3^ for the long-term studies. Mice were subjected to either 12.5 mg/kg AZD4547 (Selleckchem, S2801) or 100 mg/kg BLU9931 (Selleckchem, S7819) treatment by oral gavage. Vehicle control mice were given 1% (v/v) Tween-80 with 0.5% (w/v) carboxymethylcellulose.

For the short-term AZD4547 (ST AZD) study, mice were dosed once a day for 5 d and harvested 6 h after the last dose. Some mice were harvested at 4 d of treatment due to the tumor shrinking rapidly. For the long-term AZD4547 (LT AZD) study, mice were dosed once a day for 28 d and harvested 6 h after the last dose. For the short-term BLU9931 (ST BLU) study, mice were dosed twice a day for 5 d and harvested 6 h after the last dose. For the long-term BLU9931 (LT BLU) study, mice were dosed twice a day for 5 d, then 2 d without drug, weekly for 4 weeks. Mice were euthanized using isoflurane with cervical dislocation. The tumors were resected, diced and processed by either snap freezing in liquid nitrogen or fixing in 10% neutral buffered formalin solution for subsequent paraffin embedding.

### Patient-derived xenograft lysate preparation

PDX samples were homogenized in tubes containing zirconia beads (Biospec) and RIPA buffer supplemented with additives, using a bead ruptor 12 homogenizer (Omni International). Fully homogenized PDX samples were collected and clarified by centrifugation at 21,130 × *g* at 4 °C for 10 min, then the protein concentration was determined using a Pierce BCA protein assay kit (Thermo Scientific) according to the manufacturer’s protocol.

### Whole exome sequencing, RNA sequencing and SNP arrays

Genomic DNA and total RNA were isolated from PDX KCC_P_4043 using a genomic DNA purification kit (Promega) and a RNeasy mini kit (Qiagen), respectively. Both DNA and RNA were quantified using a Nanodrop ND-1000 (NanoDrop Technologies). DNA and RNA were dried down into specialized DNA and RNA tubes and shipped at room temperature to GENEWIZ, Suzhou, China, for sequencing. Extracted DNA of the selected PDX was sent to Ramaciotti Centre for Genomics for axiom UK biobank SNP array analysis. Copy number analysis was performed using Axiom analysis suite (v5.01.38) copy number discovery workflow. The output files from Axiom analysis suite were visualized in IGV software.

### RNA isolation, RT-PCR and Sanger sequencing

Total RNA was isolated from PDX KCC_P_4043 with a RNeasy mini kit (Qiagen) following the manufacturer’s protocol and quantified using a Nanodrop ND-1000 (NanoDrop Technologies). RNAs were reverse transcribed using a high-capacity cDNA reverse transcription kit (Thermo Fisher Scientific). Subsequently, cDNA was PCR amplified to confirm the fusion of FGFR2 and SKI using forward primers to FGFR2 exon 10 (AACAACACGCCTCTCTTCAACG), 11 (GTTGCTTTGGGCAAGTGGTC) or 12 (CTTCTTGGAGCCTGCACACA) and reverse primers to SKI exon 2 (TTTTGGGTCTTATGGAGGCCG, CTTGTCCTTTTCGGAAGGCG, AGCCCAGGCTCTTATTGGAA). The PCR products were resolved by gel electrophoresis, and the bands at the predicted product size were excised and purified with a gel and PCR clean-up system (Promega) for Sanger sequencing by the Micromon facility at Monash University. Reactions were repeated on four biological replicates.

### Derivation of human breast cancer organoids

Breast cancer tissue was cut into 1 mm fragments and digested for 90 min at 37 °C on an orbital shaker with 2.5 mg/ml collagenase (Sigma, 10103586001) in advanced DMEM/F12 (Gibco, 12634028) containing 1X Glutamax (Gibco, 35050061), 10 mM HEPES (Gibco, 15630080) and 50 µg/ml Primocin (InvivoGen, ant-pm-1) (adDF +). Tissue fragments were mechanically dissociated by repetitive pipetting with a 10 ml pipette and then a 5 ml flamed and thinned glass pipette. The resuspended cell solution was strained through a 100um cell strainer (Corning, 352360). After blocking with 2% fetal bovine serum (Gibco, 10270-106), the solution was centrifuged at 1500 rpm at 4 °C for 5 min. The cell pellet was washed with adDF + and centrifuged again at 1500 rpm at 4 °C for 5 min. In case of a visible red cell pellet, erythrocytes were lysed in 1 ml red blood cell lysis buffer (Merk, 11814389001) for 5 min at room temperature before washing with 10 ml AdDF + and centrifuging at 1500 rpm at 4 °C for 5 min. The cell pellet was then resuspended in growth factor reduced Matrigel (Corning, 356231). Matrigel containing breast cancer cells was seeded into 24-well tissue culture plates (Nunc, 142475) and allowed to polymerize for 10 min at 37 °C. The Matrigel was then overlaid with 500 µl of culture medium composed of adDF + supplemented with 1X B27 (Gibco, 17504044), 5 ng/ml recombinant human EGF (PeproTech, AF-100-15), 5 ng/ml FGF7 (PeproTech, 00-19-100), 20 ng/ml FGF10 (PeproTech, 100-26-100), 5 nM Neuregulin 1 (PeproTech, 100-03-100), 50 ng/ml IGF (BioLegend, 590908), 500 nM A83-01 (Tocris Bioscience, 2939), 1.25 mM N-acetylcysteine (Sigma, A9165), 5 mM nicotinamide (Sigma, N0636), 10% Noggin conditioned media and 10% R-spondin1 conditioned media. Following initial seeding of the cultures, 5 µM Y-27632 dihydrochloride kinase inhibitor (MedChemExpress, HY-10583) was also added to the media for 2–3 d. Organoids were maintained in a 37 °C humidified atmosphere under 5% CO_2_. The culture medium was replaced with fresh medium every 2–3 d.

### Organoid passaging

Organoids in Matrigel were mechanically scraped and collected into a tube with cold advanced DMEM/F12 (Gibco). Organoids were then centrifuged at 1500 rpm at 4 °C for 5 min and medium removed. The cell pellet was resuspended with TrypLE Express (Thermo Fisher) and incubated at 37 °C for 6 min, followed by the addition of advanced DMEM/F12 and centrifugation at 1500 rpm at 4 °C for 5 min. The supernatant was removed, and the cell pellet resuspended in growth factor reduced Matrigel and 50 μL seeded per well in a 24-well plate. After the Matrigel polymerized at 37 °C for 10 min, the Matrigel was overlaid with complete culture medium as described previously. Organoids were maintained in a 37 °C humidified atmosphere under 5% CO_2_. Culture medium was replaced with fresh complete medium without Y-27632 every 2 d after passaging.

Following the establishment of breast cancer organoids in 24-well plates, organoids were dissociated using TrypLE Express solution (Gibco) and seeded as single cells in Matrigel into a 96-well plate in triplicate. Organoids were cultured in complete medium for 3 d until small organoids formed. Reference viability values were measured at day 0 by adding 100 μL of 1 × Presto Blue reagent (Invitrogen) diluted in Advanced DMEM-F12 medium (Invitrogen) to each well. Organoids were cultured for 45 min at 37 °C before the Presto Blue solution was transferred into a black microplate (NUNC) and the fluorescence measured (excitation of 560 nm and emission of 590 nm) using the OPTIMA microplate reader (BMG Labtech). Complete medium supplemented with 10 µM BLU9931 was added to the organoids at day 0, 2 and 4. Organoid viability was measured at day 2, 4 and 6, as for day 0.

### Immunoblotting

Protein lysates were prepared in 5 × sample loading buffer (9% (v/v) glycerol, 0.03 M Tris/HCl pH 6.8, 2% (w/v) SDS, 0.05% (v/v) β-mercapethanol and 0.002% (w/v) bromophenol blue) and boiled for 10 min at 96 °C. Western blot analysis was performed by SDS-PAGE on 4% (w/v) stacking gels and 8% (w/v) separating gels. Resolved proteins were subsequently wet transferred onto PVDF membrane for 1 h, then blocked using 5% (w/v) BSA/TBS blocking buffer for 1 h at RT, followed by incubation in primary antibody diluted in 5% (w/v) BSA/TBS rolling overnight at 4 °C. Membranes were washed thrice with TBS-T for 10 min, then probed with secondary antibody for 1 h at RT. Membranes were washed thrice again for 10 min with TBS-T before signal detection by ECL (Perkin Elmer) or Luminata Forte Western HRP substrate (Millipore) and images acquired with the ChemiDoc Touch Imaging system (Bio-Rad).

The following antibodies were purchased from Cell Signaling Technology: FGFR1 (9740), FGFR2 N-terminal (23328), AKT (4685), ERK (4695), pAKT (S473) (4058), pERK (T202, Y204) (4370) and PARP (9546). The following antibodies were purchased from Santa Cruz Biotechnology: FGFR2 C-terminal (sc-6930), FGFR3 (sc-13121), FGFR4 (sc-136988) and β-actin (sc-69879). An α-tubulin antibody (T5168) was purchased from Sigma-Aldrich.

### Immunohistochemistry

Formalin-fixed paraffin-embedded (FFPE) blocks from PDX tumors or breast cancer organoids were sectioned at 4 µm onto Superfrost Plus slides. Immunohistochemistry was carried out using the DAKO Autostainer Link 48. Sections underwent dewaxing, heat-induced antigen retrieval using DAKO Target Retrieval Solution (S1699) at 98 °C for 30 min, endogenous peroxidases were quenched by applying Dako Real Peroxidase Blocking solution (S2023) for 10 min, followed by Dako Serum Free Protein Block (X0909) for 30 min. Then, primary antibody incubation using FGFR4 (sc-136988, Santa Cruz Biotechnology, 1:300 dilution) or Ki-67 antibody (9027, Cell Signaling Technology, 1:300 dilution) was followed by the Dako Envision + System – HRP Labelled Polymer Anti-Rabbit (K4003) secondary antibody incubation system. Lastly, sections were counterstained with Dako Automation Hematoxylin Histological Staining Reagent (S3301). For the Ki67 staining analysis, at least 10 field of vision images per sample were taken with ImageScope viewer and positive areas quantified using the ImageJ software. For FGFR4, the sample was considered positive if more than 1% of cells exhibited staining.

### Interrogation of breast cancer datasets and survival analysis

Breast cancer subtypes in the METABRIC and TCGA cohorts were assigned based on PAM50 + claudin-low subtype gene expression signatures. Copy number variation profile and associated overall survival data from 2509 breast cancer patients as part of the METABRIC trial were downloaded from the cBioPortal for Cancer Genomics portal (https://www.cbioportal.org/). Breast cancer patients were either not subtyped or subclassified into Basal or Luminal A, and then further classified by FGFR4 expression status into three groups exhibiting either low, normal or high expression of FGFR4 based on the 33% quantile; or by FGFR4 copy number variation (CNV) event into two groups each having either amplified copy number (FGFR4 Amp) or neutral (FGFR4 Neutral). Information regarding copy number variation (CNV) was provided from the METABRIC data where − 2 = homozygous deletion; − 1 = hemizygous deletion; 0 = neutral / no change; 1 = gain; 2 = high-level amplification.

Consequently, ‘amplified’ tumors exhibited a CNV value of 1 or 2 and were ‘neutral’ when the value was 0. Survival analyses comparing overall survival between the ‘amplification’ and ‘neutral’ subgroups, and ‘high’ and ‘low’ subgroups were subsequently performed using a Log-rank test (with *p* < 0.05 considered significant). The Log-rank test statistics and survival curves were generated using Kaplan–Meier estimate and implemented using the Logrank package (Cardillo, 2020), in MATLAB 2019b.

### Quantification and statistical analysis

Immunohistochemistry quantification was performed using NIS-Elements Viewer 4.50 and ImageJ. Quantification of Western blots by densitometry was performed using ImageLab version 5.2.1 (Bio-Rad). Statistical significance (considered significant at *p* < 0.05) was determined using either a two-way ANOVA test or an unpaired t test, as specified in the figure legend. Presence of statistical outliers was evaluated by a Grubbs’ test.

### Animal and human ethics approvals

All animal procedures were carried out in accordance with relevant national and international guidelines and animal protocols approved by the Garvan/St Vincent’s Animal Ethics Committee (Animal ethics number 15/10).

Studies on PDO were conducted in accordance with the Declaration of Helsinki, and the protocol was approved by the Cabrini Human Research Ethics Committee (CHREC 05-26-03-18) and the Monash Human Research Ethics Committee (MHREC 2018-13673-18220). Breast tumors were obtained from treatment naïve breast cancer patients undergoing surgical resection at Cabrini Health, Brighton, Australia. All subjects provided written informed consent.

IHC studies on primary breast cancer specimens were conducted in accordance with the NHMRC Statement on Ethical conduct in Human Research and were approved by the Alfred Health Ethics Committee (Project Number 598/18). Tissue was obtained from patients diagnosed with breast cancer from Alfred Health Anatomical Pathology department. Breast cancers were classified as luminal (12 cases) or TNBC (14 cases) based on the positive ER and PR expression and negative ER, PR and HER2 expression, respectively. For the luminal cancers, 9 cases were invasive NOS, 1 case of mucinous carcinoma and 2 cases of invasive lobular carcinoma. A HER2-amplified case was also included.

## Results

### Expression and phosphorylation of FGFRs in breast cancer PDXs

**Fig. 1:**
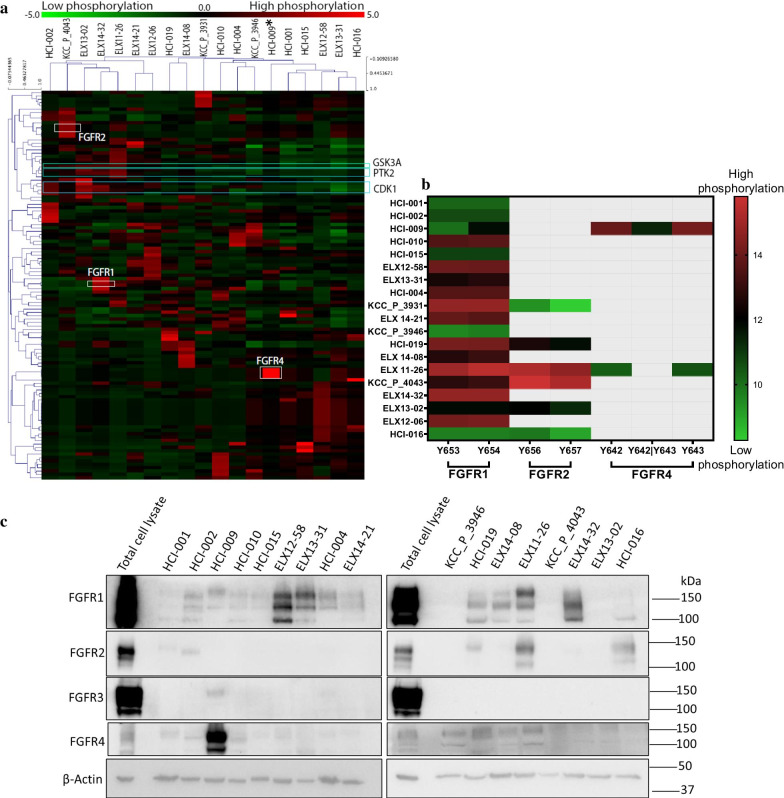
FGFR expression and phosphorylation signatures in human breast cancer PDX as determined by MS-based tyrosine phosphorylation profiling and immunoblotting. **a** Unsupervised hierarchical clustering of PDX based on 115 identified tyrosine-phosphorylated kinase peptides. Relative abundance is based on z-score across the 19 PDX samples. The luminal-B PDX HCI-009 is indicated by an asterisk. **b** Site-selective phosphorylation of specific FGFRs based on z-score across the 19 PDX samples. Gray shading indicates that the FGFR phosphorylation site was undetectable by MS. **c** Expression of specific FGFRs across the panel. Protein lysates from 17 PDX samples were immunoblotted with the indicated antibodies. Total cell lysate indicates lysates from specific TNBC cell lines used as positive controls for the respective antibodies (CAL120 for FGFR1, MFM-223 for FGFR2, SUM185PE for FGFR3 and MDA-MB-453 for FGFR4)

In order to identify potential therapeutic targets, global MS-based phosphotyrosine profiling was conducted across a panel comprising 18 TNBC and 1 luminal B PDX. A total of 897 tyrosine phosphorylated peptides were identified that included 115 kinase-derived peptides. The latter were subjected to unsupervised hierarchical clustering and ranking according to combined kinase or individual phosphopeptide intensity (Fig. 1a, Additional file 1: Fig. S1, Additional file 2: Tables S1–S2). Clustering resolved two major subgroups, with one characterized by elevated tyrosine phosphorylation of CDK1, GSK3A and PTK2 (Fig. 1a). Also evident in the heat map was pronounced phosphorylation of specific FGFRs in particular PDX, including KCC_P_4043 (FGFR2), ELX14-32 and ELX11-26 (FGFR1) and HCI-009 (FGFR4). Of note, certain kinases (e.g., GSK3A, DYRK1A, CDK1) exhibited relatively high levels of tyrosine phosphorylation across all the PDX, while others represented ‘outlier’ kinases in only one or two PDX models (e.g., FGFR4, MAPK8, MAPK9) (Additional file 2: Tables S1–S2). Assessment of ‘outlier’ kinases using a Z-score approach revealed that some PDX exhibited only one outlier (e.g., FGFR4 in HCI-009) while others featured many (e.g., 18 in ELX12-58) (Additional file 3: Fig. S2). Consequently, the landscape of kinase tyrosine phosphorylation across the PDX panel was complex and exhibited considerable heterogeneity.

Since elevated tyrosine phosphorylation of particular FGFRs was observed for several PDX, and these RTKs are implicated in breast cancer development and progression and represent therapeutic targets, data relating to FGFR site-selective phosphorylation were extracted from the dataset for further interrogation (Fig. 1b). This revealed PDX with relatively high phosphorylation of both FGFR1 and FGFR2 (ELX11-26), FGFR2 alone (KCC_P_4043) and FGFR4 alone (HCI-009). In order to characterize FGFR expression, 17 breast cancer PDX samples were subjected to Western blot analysis using selective FGFR antibodies (Fig. 1c). In general, the results were concordant with the tyrosine phosphorylation data, indicating that increased FGFR phosphorylation was associated with elevated expression. Thus, while most PDX exhibited detectable FGFR1 expression, FGFR1 overexpression was observed in ELX12-58, ELX13-31, ELX14-32 and ELX11-26, and robust FGFR4 expression was only detected in HCI-009. However, while strong expression of FGFR2 was detected in ELX11-26, the FGFR2 C-term antibody did not detect a band of predicted size in KCC_P_4043 lysate, despite this PDX exhibiting the highest relative FGFR2 phosphorylation. This issue is revisited later in the manuscript.

### Selective inhibition of FGFR1-3 in PDX models of TNBC using AZD4547

Given their high phosphorylation of FGFR1 and/or FGFR2 (Fig. 1b), two TNBC PDX, ELX11-26 and KCC_P_4043 were selected for treatment with a selective FGFR1-3 inhibitor, AZD4547 [36]. A further TNBC PDX, HCI-016, was chosen as a negative control given detectable FGFR1 and FGFR2 expression but low receptor phosphorylation (Fig. 1b, c).

**Fig. 2:**
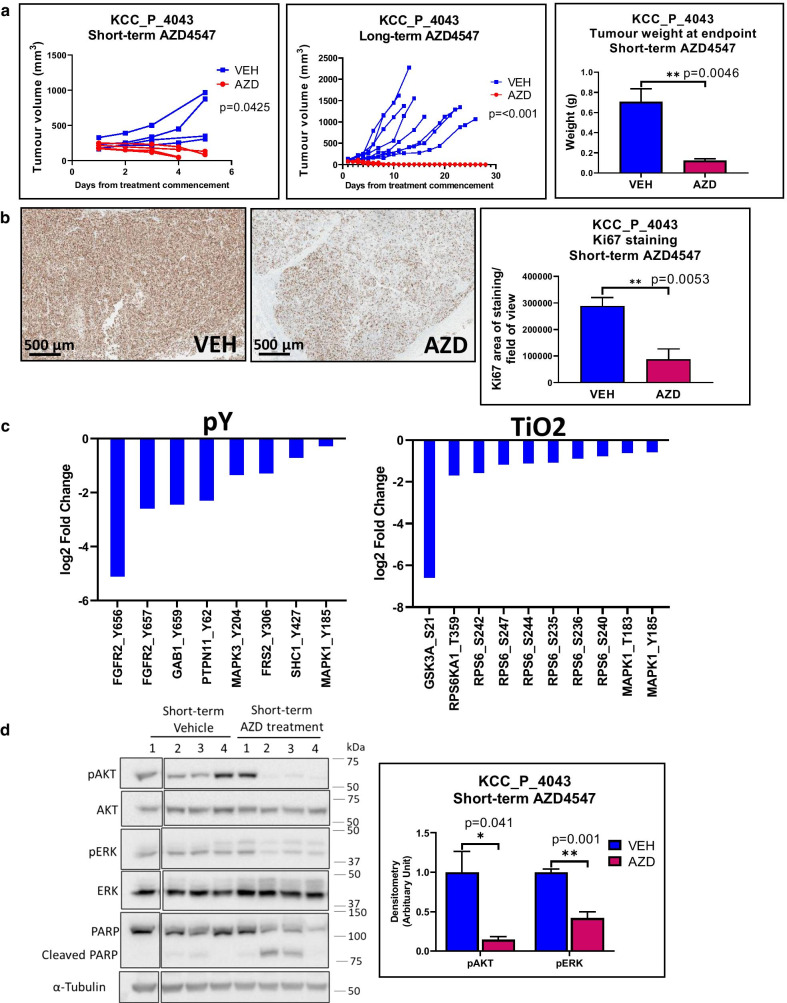
Effect of FGFR1-3 inhibitor AZD4547 on the KCC_P_4043 PDX model. **a** Effect on tumor growth. Mice were treated with vehicle control or AZD4547 for short term (5 d; 4 mice per group) or long term (28 d; 10 mice per group) and the tumor volume measured daily. Statistical significance for short-term treatment group was determined using the two-way ANOVA test (*p* value = 0.0425). Statistical significance for long-term treatment group was determined by unpaired t test at Day 14 (*p* value < 0.001). Tumor weight at endpoint for the short-term treatment group was also determined, with statistical significance determined by unpaired t test. **b** Effect on cell proliferation. FFPE tumor sections from the short-term treatment group were stained for Ki67 and quantified. Statistical significance was determined using the unpaired t test. **c** Effects on site-selective protein phosphorylation as determined by MS-based phosphoproteomics. Data from phosphotyrosine- and TiO2-enrichment workflows are presented, highlighting phosphosites downregulated in response to AZD4547. **d** Effects on downstream signaling determined by Western blotting. Lysates were Western blotted as indicated. Phosphorylated AKT and ERK were quantified by densitometry. Data were first normalized relative to the tubulin control, then phosphorylated proteins normalized to the total protein and expressed relative to the average of the vehicle control which was arbitrarily set at 1.0. Mouse 1 of the AZD4547 treatment group was excluded from this analysis due to ineffective drug delivery. Statistical significance was determined by unpaired *t* test. * indicates *p* value of < 0.05, ** < 0.01. Error bars: mean ± standard error of biological replicates

Out of the three TNBC PDX, KCC_P_4043 demonstrated high sensitivity to AZD4547 (Fig. 2). This PDX was originally derived from a TNBC primary tumor and is a very fast-growing. No macroscopic metastases were observed with this model, which is common for rapidly growing tumors, as metastatic progression does not have time to occur. AZD4547 treatment significantly reduced tumor volume in both the short and long-term treatment groups and also significantly reduced tumor weight at endpoint in the former group, while tumors in the long-term AZD4547 group were eliminated (Fig. 2a). Tumor sections stained for Ki67 revealed that short-term AZD4547 treatment significantly inhibited cell proliferation compared to the vehicle control (Fig. 2b). In order to confirm on-target activity of the drug and characterize its effect on downstream signaling in an unbiased fashion, PDX lysates from control and drug-treated mice were subjected to global phosphoproteomic profiling using both TiO_2_ and phosphotyrosine-enrichment workflows (Additional file 5: Table S3). Site-selective phosphorylation of proteins related to the FGFR signaling pathway (including FGFR2 and FRS2), PI3K/AKT (GSK3A) and RAS/MAPK (SHC1, PTPN11, MAPK3, MAPK1) was markedly decreased by AZD4547 treatment, as was phosphorylation of RPS6KA1 and RPS6, proteins implicated in controlling cell growth (Fig. 2c). Inhibition of AKT and ERK was confirmed by Western blotting, which also revealed enhanced PARP cleavage in drug-treated PDX (Fig. 2d). Bioinformatic analyses of regulated phosphosites revealed enrichment for kinase pathways associated with RTK, cytokine and adhesion signaling, but also an unexpected and marked effect on the ‘spliceosome’ (Additional file 6: Table S4). RNAseq analysis revealed an additional effect of the drug on gene expression relating to cell metabolism, specifically glycolysis/gluconeogenesis and fructose and mannose metabolism, and also extracellular matrix organization (Additional file 6: Table S4). Overall, these data highlight on-target FGFR2 inhibition and decreased mitogenic, growth and survival signaling in KCC_P_4043 PDX upon AZD4547 treatment, and also novel downstream effects of this drug. In ELX11-26 and HCI-016, AZD4547 had no significant effect on tumor volume and tumor weight at endpoint (Additional file 4: Fig. S3). These results indicate that high FGFR phosphorylation does not always confer sensitivity to FGFR inhibition and highlights the need for additional predictive biomarkers.

### TNBC PDX KCC_P_4043 harbors a novel FGFR2-SKI fusion

**Fig. 3:**
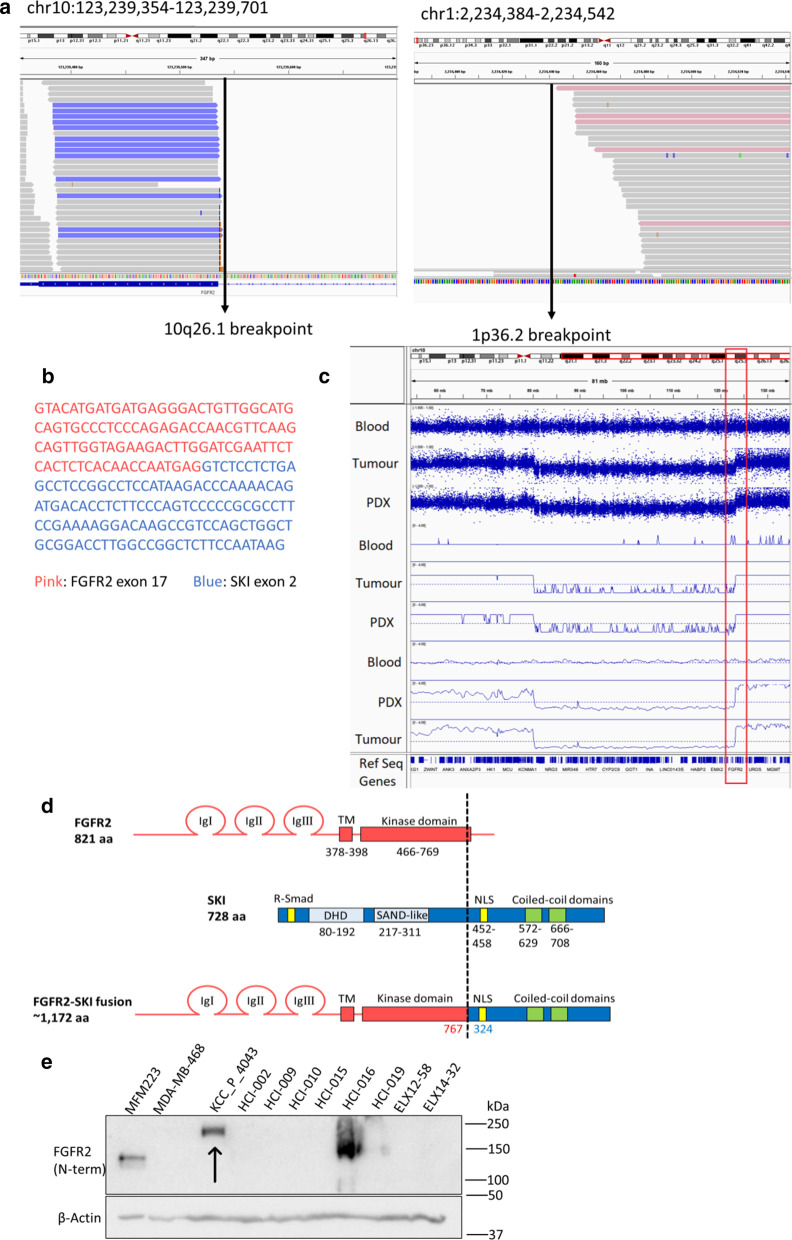
Characterization of the FGFR2-SKI fusion identified in the KCC_P_4043 PDX model. **a** Integrative Genomic Viewer results for breakpoint regions of chromosome 10 (containing FGFR2) and chromosome 1 (SKI). **b** Junction break point sequence of FGFR2-SKI fusion. Pink, FGFR2 exon 17; blue, SKI exon 2. **c** Analysis of FGFR2 in the KCC_P_4043 using SNP arrays. Top three tracks are copy number raw logRatio data from the array; middle three tracks are copy number segmentation from the raw logRatio; bottom three tracks are smoothed copy number signal. The red box highlights FGFR2 on chromosome 10. **d** Schematic of the FGFR2-SKI fusion in KCC_P_4043. Domain structure and amino acid residues of FGFR2 and SKI are indicated. In FGFR2, IgI–IgIII: immunoglobulin 1–3. TM: transmembrane domain. In SKI, R-smad: corresponding binding domain. DHD: Dachshund homology domain. SAND: Sp100, AIRE1, NucP41/75 and DEAF1. NLS: nuclear localization sequence. The dotted line highlights the junction between FGFR2 and SKI. **e** Confirmation of FGFR2-SKI expression by Western blotting. Protein lysates from 9 PDX samples were immunoblotted with a FGFR2 N-term antibody. MFM-223 and MDA-MB-468 lysates were used as positive and negative controls, respectively

High FGFR2 phosphorylation and AZD4547 dependency suggested a possible oncogenic form of FGFR2 in KCC_P_4043, which led us to conduct whole exome sequencing (WES) and RNAseq analyses. WES analysis detected 367 gene mutations, mostly non-synonymous SNVs and no mutations in the FGFR family in the KCC_P_4043 model (Additional file 7: Table S5). A predicted splicing alteration in the tumor suppressor gene BRCA2 and a R294C mutation in centrobin, a centrosomal BRCA2 interacting protein were detected, which may have contributed to cancer progression in this model (Additional file 7: Table S5). Other mutations associated with DNA/RNA replication (POLA1, POLR1A), the spliceosome (PRPF40A), signaling pathways (insulin, Wnt, mTOR, MAPK, ErbB, phosphatidylinositol), specific phosphatases (PPP1R3D, PLD2 and PPM1A), glycolysis/gluconeogenesis (ADPGK) and fructose and mannose metabolism (ketohexokinase) were also detected (Additional file 7: Table S5). Gene fusion analysis using the RNA sequencing data revealed a junction breakpoint involving FGFR2 and SKI on chromosome 10 and 1, respectively (Fig. 3a). Alignment of the junction breakpoint reads to the FGFR2 and SKI templates revealed that the breakpoint occurs at FGFR2 exon 17 and SKI exon 2 (Fig. 3b). These results suggest a chromosomal translocation event t(10;1)(q26.1;p36.2) in KCC_P_4043. This interpretation was further supported by use of SNP arrays that identified a breakpoint in the FGFR2 gene with increased copy number toward the 5ʹ end, that was specific to PDX and tumor, and not detected in the matching patient’s blood sample (Fig. 3c). To confirm the presence of a FGFR2-SKI fusion in KCC_P_4043, we designed 3 sets of PCR primers targeting FGFR2 exons 10 to 12 (forward primers) and SKI exon 2 to 3 (reverse primers) (Additional file 4: Fig. S4a) to directly detect the fusion by reverse-transcription PCR (RT-PCR) using RNA from this PDX. Amplified PCR products of the predicted sizes were identified (Additional file 4: Fig. S4b), and sequencing confirmed the FGFR2-SKI fusion transcript containing the majority of the FGFR2 kinase domain (two C-terminal amino acids, glutamate and tyrosine were deleted) (Additional file 4: Fig. S4c–d).

Previously, FGFR2 expression could not be detected in KCC_P_4043 lysate using the FGFR2 C-term antibody (Fig. 1c). This is explained by the gene fusion event, that removes the C-terminal region of FGFR2 (Fig. 3d). Indeed, Western blotting with an antibody raised against the N-terminal region of FGFR2 detected a band at approximately 200 kDa, a lower mobility than endogenous FGFR2 in FGFR2-amplified MFM-223 breast cancer cells and the PDX HCI-016 (140–150 kDa) (Fig. 3e). Allowing for the known glycosylation of FGFRs, and the phosphorylation-induced gel retardation of SKI through phosphorylation at S515 [37], this gel mobility is consistent with that expected for the FGFR2-SKI fusion. For PDX ELX11-26, SNP arrays demonstrated the presence of an amplicon spanning the entire FGFR2 gene (Additional file 4: Fig. S5), explaining the high expression of FGFR2 in this PDX (Fig. 1c). However, despite high FGFR1 phosphorylation in this PDX, the FGFR1 gene was not amplified (Additional file 4: Fig. S6).

### Selective inhibition of FGFR4 in a PDX model of luminal B breast cancer using BLU9931

**Fig. 4:**
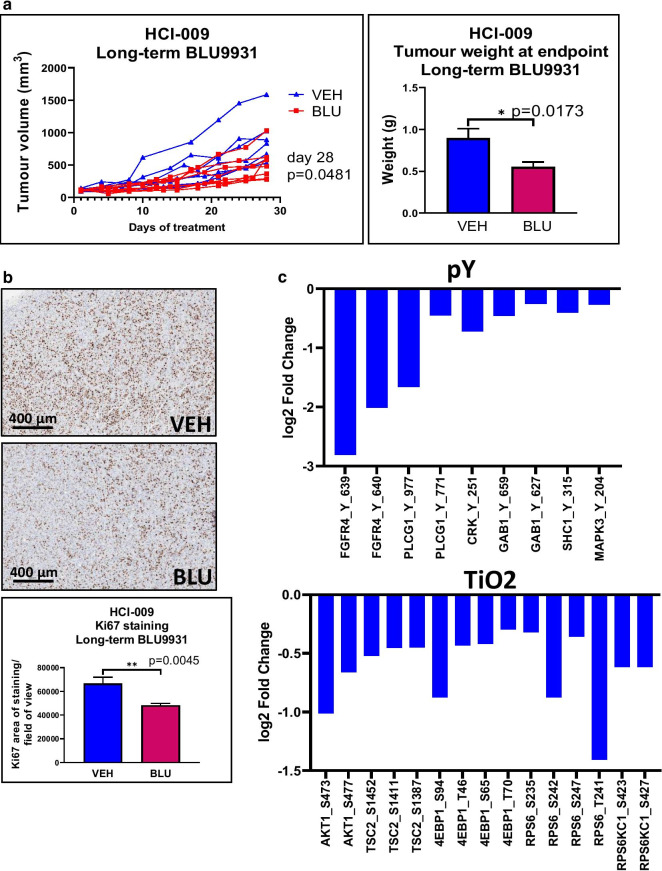
Effect of FGFR4 inhibitor BLU9931 on the HCI-009 PDX model. **a** Effect on tumor growth. Mice were treated with vehicle control or BLU9931 for long term (28 d; 7 mice in Vehicle group, 8 mice in BLU group) and the tumor volume measured daily. Statistical significance was determined using an unpaired t test at endpoint (Day 28, *p* value 0.0481). Absence of statistical outliers was confirmed by a Grubbs’ test. Tumor weight at endpoint for the long-term treatment group was also measured with significance determined by an unpaired t test. Absence of statistical outliers was confirmed by a Grubbs’ test**. b** Effect on tumor cell proliferation. FFPE tumor sections from the long-term treatment group were stained for Ki67 and the data quantified. Statistical significance was determined using an unpaired t test. Use of a Grubbs’ test detected one outlier in the BLU-treated group, *p* value with outlier = 0.0218, *p* value with outlier removed = 0.0045. * indicates *p* value of < 0.05, ** < 0.01. Error bars: mean ± standard error of biological replicates. **c** Effects on site-selective protein phosphorylation as determined by MS-based phosphoproteomics. Data from phosphotyrosine- and TiO2-enrichment workflows are presented, highlighting phosphosites downregulated in response to BLU9931

The HCI-009 PDX model exhibiting high FGFR4 expression and phosphorylation was subjected to treatment with a FGFR4 inhibitor, BLU9931 [15] to characterize effects on tumor growth in vivo, cell proliferation and FGFR4 downstream signaling (Fig. 4). This PDX was established from a TNBC ascites and is a relatively slow-growing model with low metastatic potential, with macroscopic metastases observed in only 2 mice out of 116. Metastases were observed in the draining lymph node and lung. Long-term BLU9931 treatment significantly decreased tumor volume, tumor weight at endpoint and decreased cell proliferation within tumors as assessed by Ki67 staining (Fig. 4a, b). MS-based phosphoproteomic analysis was used to determine the effect on downstream signaling (Fig. 4c). FGFR4 phosphorylation at Y639 displayed the largest decrease in the phosphorylated tyrosine enrichment dataset, confirming efficient FGFR4 targeting by BLU9931 (Fig. 4c, Additional file 8: Table S6). Downstream targets of FGFR4 signaling, including PLCG1, GAB1 and AKT also exhibited reduced phosphorylation (Fig. 4c). Bioinformatic analyses revealed similarities with the effects of AZD4547 on the TNBC PDX, particularly on tyrosine kinase-regulated pathways, but impact of BLU9931 on the spliceosome was less evident than with AZD4547, and other pathways affected by BLU9931 included central carbon and fatty acid biosynthesis, RIG-I-like and NOD-like receptor signaling, and apoptosis (Additional file 9: Table S7). Interestingly, despite the marked overexpression of FGFR4 in HCI-009, the FGFR4 gene was not amplified in this PDX, as determined by SNP arrays (Additional file 4: Fig. S7).

### Interrogation of FGFR2 and FGFR4 alterations in breast cancer patients using public datasets

**Fig. 5:**
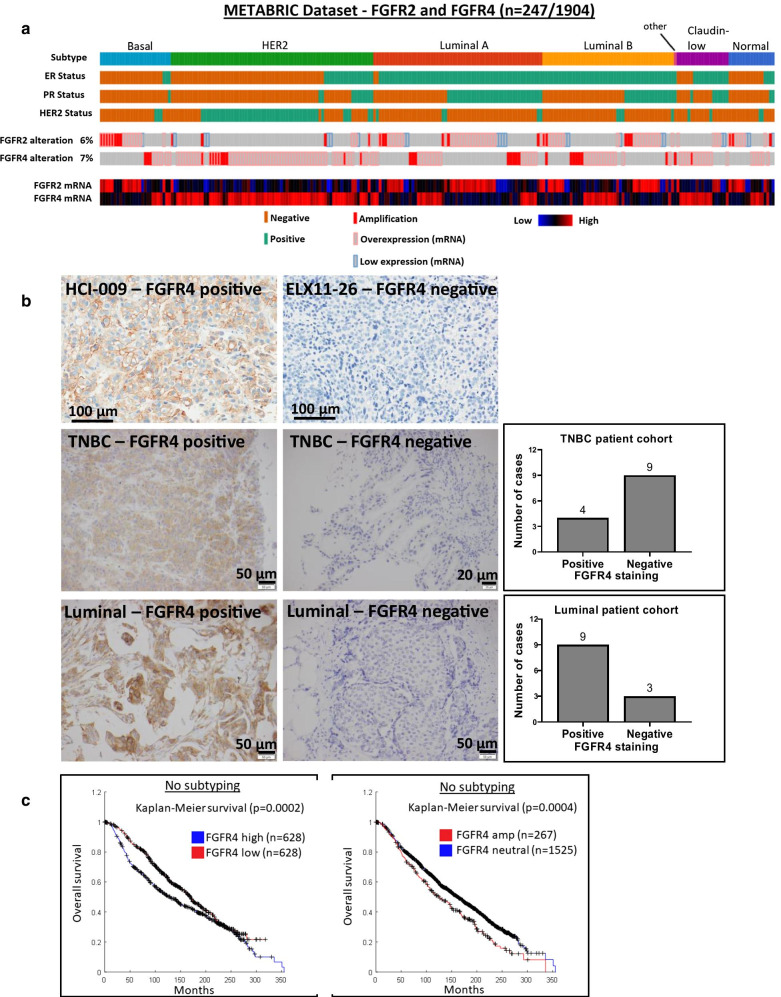
FGFR2 and FGFR4 alterations in breast cancer patients. **a** Frequency of FGFR2 and FGFR4 alterations in different breast cancer subtypes. Data were extracted from the METABRIC dataset in cBioPortal. Only patients with FGFR alterations are displayed for brevity. **b** Immunohistochemical staining for FGFR4 on breast cancer specimens. PDX HCI-009 and ELX11-26 were used as positive and negative controls, respectively. A cohort of 12 luminal breast cancer and 13 TNBC samples were stained for FGFR4 expression. The frequency of positive and negative staining in these cohorts is represented in the bar graphs. **c** Association of FGFR4 alterations with patient prognosis. Kaplan–Meier plots using data from the METABRIC dataset indicating that patients with FGFR4 overexpression (left panel) or amplification (right panel) exhibit worse overall survival compared to those without FGFR4 alteration. A Logrank test was used where a *p* value of < 0.05 was considered significant. Survival data for the different patient groups were extracted and downloaded from cBioPortal and survival analysis performed using an in-house Matlab script

Our data highlighting effective targeting of oncogenic FGFR2 and FGFR4 alterations in specific PDX led us to analyze the METABRIC and TCGA datasets using cBioPortal to determine the frequency of FGFR2 and FGFR4 genomic and expression changes in different breast cancer subtypes (Fig. 5a, Additional file 4: Fig. S8a). In the METABRIC dataset, 247 out of 1904 (13%) breast cancer patients have FGFR2 (6%) and/or FGFR4 (7%) alterations (Fig. 5a). In the TCGA dataset, 126 out of 994 (13%) breast cancer patients have FGFR2 (7%) and/or FGFR4 (6%) alterations (Additional file 4: Fig. S8a).

FGFR2 amplification, which occurred in 26 (1.4%) and 15 (1.5%) breast cancer patients in the METABRIC and TCGA datasets, respectively, was observed in all subtypes except the claudin-low subtype (Fig. 5a, Additional file 4: Fig. S8a). FGFR2 overexpression occurred in both the TNBC/basal and luminal subtypes and was rarely observed in HER2 cancers (Fig. 5a, Additional file 4: Fig. S8a), while 19 breast cancer patients (1.9%) exhibited mutation or fusion of FGFR2 in the TCGA dataset (Additional file 4: Fig. S8a).

FGFR4 amplification occurred in 30 (1.6%) and 13 (1.3%) breast cancer patients in the METABRIC and TCGA datasets, respectively, and was observed in all subtypes, except normal (Fig. 5a, Additional file 4: Fig. S8a). FGFR4 overexpression was mostly detected in the HER2 subtype, followed by the luminal subtypes (Fig. 5a, Additional file 4: Fig. S8a). Only 5 patients exhibited FGFR4 mutation in the TCGA dataset, with no fusions reported (Additional file 4: Fig. S8a). To complement these analyses, FFPE tissue sections from luminal and TNBCs were subjected to IHC staining for FGFR4, with conditions optimized using HCI-009 and ELX11-26 PDX as positive and negative controls, respectively (Fig. 5b). Approximately one-third (4 out of 13) of the TNBC specimens exhibited FGFR4 positivity, while the majority (9 out of 12) of luminal samples scored positive (Fig. 5b). We also detected FGFR4 positivity in a HER2-amplified case (Additional file 4: Fig. S8b).

In the METABRIC dataset, breast cancer patients (no particular subtyping) with high FGFR4 expression or amplified FGFR4 exhibited a significantly worse overall survival compared to breast cancer patients with unaltered FGFR4 (Fig. 5c). Among breast cancer subtypes, TNBC/basal breast cancer patients with high FGFR4 expression and luminal A patients with amplified FGFR4 displayed a significantly worse overall survival (Additional file 4: Fig. S8c).

### Characterization of FGFR4 expression and function in breast cancer patient-derived organoids

**Fig. 6:**
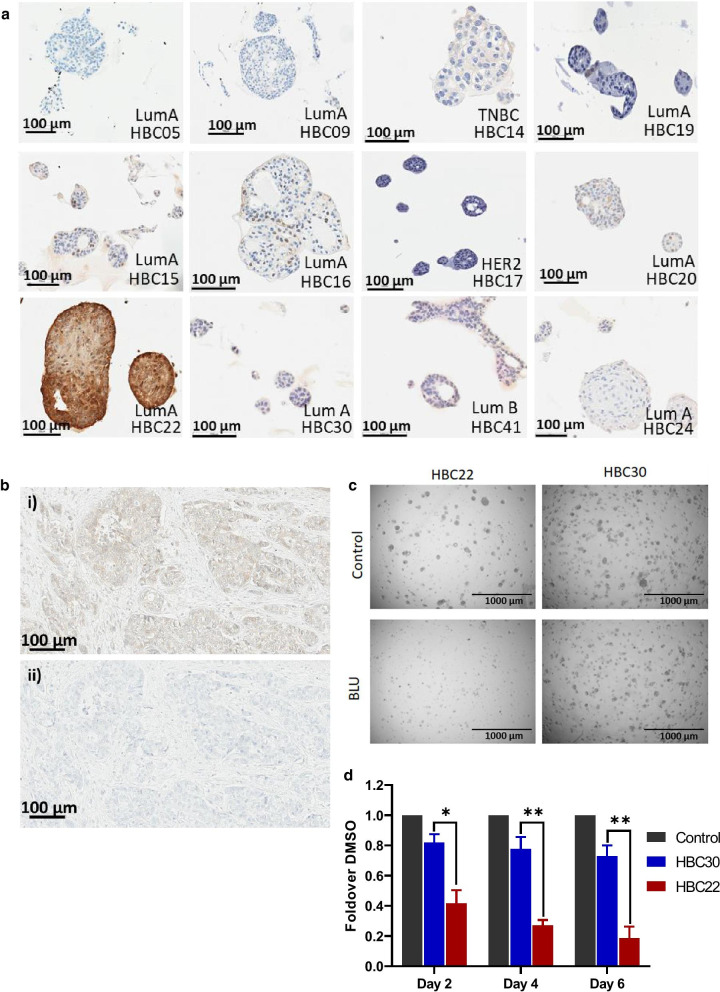
FGFR4 expression and functional characterization in human breast cancer organoids. **a** Immunohistochemical staining for FGFR4 across a panel of 9 human breast cancer organoid lines. **b** Immunohistochemical staining for FGFR4 of the original tumor tissue used to establish the HBC22 organoid line. Panel i) shows staining with the FGFR4 antibody, while ii) is the minus antibody control. **c** Effect of the FGFR4 inhibitor BLU9931 on organoid growth. Images of DMSO control or BLU9931-treated FGFR4-high HBC22 and FGFR4-low HBC30 organoids at endpoint. **d** Quantification of (c) comparing the FGFR4-high HBC22 organoid line (red) with the FGFR4-low HBC30 line (blue) normalized to the DMSO control. Statistical significance was determined using an unpaired t test. * indicates *p* value of < 0.05, ** < 0.01. Error bars: mean ± standard error of three biological replicates, each with three technical replicates

The occurrence of FGFR4 genomic and/or expression changes in particular breast cancer subtypes and the association of these changes with poor prognosis led us to further interrogate FGFR4 function using a panel of breast cancer PDOs spanning the luminal A, HER2 and TNBC subtypes. These were initially screened for FGFR4 expression by IHC (Fig. 6a). Only the luminal A HBC22 organoid line exhibited strong positive FGFR4 staining, while low or undetectable FGFR4 staining was observed in the remaining lines (Fig. 6a). This organoid line was derived from a multifocal pT2 N1, grade 3 invasive carcinoma of the breast, which also scored positive for FGFR4 expression (Fig. 6b). HBC22 was selected as a candidate line to investigate the effect of FGFR4 inhibitor BLU9931 on organoid proliferation, with HBC30 used as a negative control (Fig. 6c, d). BLU9931 significantly decreased organoid proliferation in the HBC22 line compared to the HBC30 negative control (Fig. 6c, d). These findings build upon our effective inhibition of aberrant FGFR4 signaling in the HCI-009 luminal B PDX (Fig. 4), highlighting FGFR4 expression as a potential therapeutic target in the luminal, and potentially other, breast cancer subtypes.

## Discussion

In this study, we have combined integrated, multi-omics analyses with use of powerful patient-derived models to identify aberrant FGFR signaling as a potential therapeutic target in specific breast cancer subtypes. Importantly, this approach detected a novel FGFR2 fusion and marked FGFR4 overexpression and activation that would not have been identified by WES, and determined that the corresponding PDX are sensitive to corresponding selective FGFR inhibitors. This provides further evidence that targetable FGFR fusions do occur in breast cancer, albeit at low frequency, and adds further weight to emerging evidence highlighting FGFR4 as a potential therapeutic target in this malignancy [28, 29].

An interesting finding was the identification of a novel FGFR2-SKI fusion in a TNBC PDX with marked sensitivity to AZD4547, indicating an oncogenic addiction to the fusion. Fusion partners of FGFR2 reported specifically in breast cancer are AFF3, CASP7 and CCDC6 [38, 39], while partners identified in other cancers include TACC and KIAA family members, PPHLN1, NTRK1, BICC1, AHCYL1, OFD1 and SLC45A3 [38, 40–42]. These are often fused to the C-terminal region of FGFR2, and by providing additional domains that mediate oligomerization, drive activation of the fusion receptor and ligand-independent signaling [38]. Similar to a FGFR3-TACC3 fusion in TNBC previously characterized by ourselves and others [24, 25], FGFR2 fusions only occur at a very low frequency in breast cancer. Indeed, the frequency of FGFR2 mutations or translocations in the TCGA breast cancer dataset was approximately 2% of patients. However, given the poor prognosis and paucity of targeted treatments for TNBC and the oncogenic addiction observed for the FGFR3-TACC3 and FGFR2-SKI fusion, screening for such alterations appears justified.

SKI is a proto-oncogene that was first discovered as the cellular counterpart of the transforming protein of the Sloan-Kettering avian retrovirus [43]. It can reside in the nucleus or cytoplasm and exhibits aberrant expression in a variety of cancers, but fusions involving SKI have not been reported. Its best-characterized function is as a negative regulator of the TGF-β signaling pathway, where it forms an inhibitory complex with SMAD proteins on TGF-β target gene promoters, and this complex recruits histone deacetylases and other repressors to inhibit gene transcription [43]. In the FGFR2-SKI fusion, the C-terminal region of SKI containing the coiled-coil domains is joined to the extreme end of the FGFR2 kinase domain, and the coiled-coil domains originating from SKI are likely to promote homodimerization [44]. The structure of this fusion, which contains the majority of the RTK FGFR2 and also the nuclear localization signals of SKI, raises the possibility that it may signal in the plasma membrane and/or nuclear compartment.

While the TNBC PDX model ELX11-26 exhibited high FGFR1 phosphorylation (but not gene amplification), FGFR2 gene amplification and also FGFR2 phosphorylation only slightly lower than KCC_P_4043, it did not respond to AZD4547. Since a previous clinical trial and studies using pre-clinical models have reported an association between high-level amplification of FGFR2 and response to selective FGFR1-3 inhibitors, with marked elevation of FGFR2 resulting in an oncogene addiction phenotype via transactivation of other RTKs, it is possible that the modest overexpression of FGFR1/2 in ELX11-26 does not traverse the threshold required to impart AZD4547 sensitivity [9, 11]. A further contributing factor to the resistance of this PDX to drug treatment may be the high activation of other kinases, including the RTKs EPHB1/3/4 and PDGFRA, and the cytoplasmic tyrosine kinases ABL2 and PTK2, which may make signaling by FGFR1/2 redundant. Overall this highlights the importance of identifying predictive biomarkers of response to FGFR inhibitors. Currently, high-level amplification of the genes encoding FGF19 (for FGFR4 targeting) and FGFR2 represent potential biomarkers for response to corresponding FGFR-directed therapies. In the case of FGFR2, this would be a gene to centromere ratio > 4–5 [9, 11]. The presence of specific activating mutations and fusions also appear to be predictive of response [9]. However, it is likely that other factors outside of FGFR aberrations will also determine drug sensitivity, including co-expression of other RTKs (e.g., particular members of the erbB family) and alterations in downstream signaling components (e.g., mutation of PI3K). Further interrogation of tumor specimens derived from clinical trials [11], as well as use of patient-derived models such as organoids that facilitate detailed analysis of marker expression, signaling pathway activation and multiple biological responses including cellular proliferation, apoptosis and invasion, will undoubtedly make major contributions in this area.

Importantly, our studies support a role for FGFR4 as a therapeutic target in specific breast cancer subtypes. Currently, the mechanisms underpinning FGFR4 overexpression in breast cancer are unclear. The luminal B PDX HCI-009 exhibited extremely high FGFR4 expression in the absence of gene amplification, suggesting a transcriptional or post-transcriptional mechanism, and limited correlation between FGFR4 DNA and mRNA has been noted in other studies [45]. However, both HCI-009 and the FGFR4-overexpressing luminal A PDO HBC22 exhibited significant sensitivity to the selective FGFR4 inhibitor BLU9931. In this regard, several previous studies have reported an association between FGFR4 and progression of luminal breast cancers. Specifically, enhanced expression of FGFR4 is associated with development of endocrine resistance in vitro [45] and poor outcome in tamoxifen-treated patients [46]. In addition, FGFR4 is overexpressed in metastases derived from luminal A breast cancers compared to the primary tumor [47] and high FGFR4 expression and hotspot mutations occur in endocrine therapy-treated distant breast cancer metastases, particularly derived from invasive lobular carcinoma [28]. Moreover, a recent study has determined that FGFR4 drives phenotypic switching of luminal A breast cancers to a HER2-enriched gene expression phenotype, and that a FGFR4-induced expression signature is positively associated with poor outcome and site-selective metastasis [29]. In the latter study, treatment of an ER-positive, HER2-enriched and FGFR4-positive PDX with BLU9931 resulted in marked inhibition of tumor growth [29]. Collectively, this work indicates that FGFR4 inhibitors may have significant impact in management of advanced, endocrine-resistant luminal breast cancer.

## Conclusion

This work demonstrates the power of applying an integrated, multi-omics approach to patient-derived models in order to identify potential therapeutic targets, provides further evidence that FGFR fusions, while occurring at a relatively low frequency in breast cancer, can confer oncogenic addiction and result in marked therapeutic responses to corresponding targeted therapy and highlights FGFR4 as an attractive target in a subset of advanced luminal breast cancer.

## Supplementary Information

**Additional file 1. Fig. S1.** High-resolution version of Figure 1a with individual kinases labeled on heat map.

**Additional file 2. Table S1.** Heat map ranking the total phosphopeptide intensity for specific kinases across the 19 PDX samples. **Table S2**. Heat map ranking individual phosphopeptide intensity for specific kinases across the 19 PDX samples.

**Additional file 3. Fig. S2.** Outlier kinases based on z-score of summed pY peptides of the kinase across PDX samples. Kinases with a z-score >1.5 were identified for each PDX sample and then expressed as a % of the summed Z scores.

**Additional file 4. Fig. S3.** Effect of FGFR1-3 inhibitor AZD4547 on **a** ELX11-26 and **b** HCI-016 PDX models. Mice were treated with vehicle control or AZD4547 for long term (28 d; 8 mice per group) and the tumor volume measured daily. Statistical significance was determined using an unpaired t test at the final timepoint (ELX11-26, *p* value = 0.825; HCI-016, *p* value = 0.403). Tumor weight at endpoint for the long-term treatment group was also measured and statistical significance was determined using an unpaired t test. **Fig. S4.** Identification of FGFR2-SKI fusion in KCC_P_4043.** a** Schematic of the primer pairs 1–3 targeted to the start of the FGFR2 kinase domain at exon 10 to 12 and at SKI exon 2 to 3 for PCR. Black arrows are the forward primers, gray arrows are the reverse primers. The predicted PCR product sizes are indicated. **b **FGFR2-SKI RT-PCR. The PCR products using primer pairs 1–3 from (a) were resolved by DNA gel electrophoresis and the bands were imaged using a fluorescent illuminator. **c** Sequence of the FGFR2-SKI fusion junction in KCC_P_4043 derived from RT-PCR products. **d** Amino acid sequence of the FGFR2-SKI fusion showing that the majority of the FGFR2 kinase domain (highlighted in yellow) is present in the FGFR2-SKI fusion. Only 2 amino acids, glutamate and tyrosine are missing (highlighted in green) from the FGFR2 kinase domain of the FGFR2-SKI fusion. Red, FGFR2; blue, SKI. **Fig. S5.** SNP arrays showing FGFR2 alterations in specific PDX. Track order as for Fig. 3c. 13-31 is a PDX with FGFR2 copy number gain included as a positive control. **Fig. S6.** SNP arrays showing FGFR1 alterations in specific PDX. **Fig. S7.** SNP arrays showing FGFR4 alterations in specific PDX. **Fig. S8.** FGFR2 and FGFR4 alterations in human breast cancer. **a** Frequency of FGFR2 and FGFR4 alterations in different breast cancer subtypes. Data were extracted from the TCGA Pan-cancer Atlas dataset in cBioPortal. Only patients with FGFR alterations are displayed for brevity. **b **FGFR4 positive immunohistochemical staining in a HER2-amplified breast cancer sample.** c** Association of FGFR4 alterations with patient prognosis. Kaplan–Meier plots using data from the METABRIC dataset indicating that patients with basal or luminal subtype cancers with FGFR4 overexpression (left panel) or amplification (right panel) exhibit worse overall survival compared to those without FGFR4 alteration. A Logrank test was used where *p* value of < 0.05 is considered significant. Survival data for the two patient groups were extracted and downloaded from cBioPortal and survival analysis performed using an in-house Matlab script.

**Additional file 5. Table S3.** MS data for both TiO2 and pTyr enrichment workflows applied to AZD4547-treated KCC_P_4043.

**Additional file 6. Table S4.** Bioinformatic analysis of MS data for drug-treated KCC_P_4043.

**Additional file 7. Table S5.** SNVs in KCC_P_4043 tumor.

**Additional file 8. Table S6.** MS data for both TiO2 and pTyr enrichment workflows applied to BLU9931-treated HCI-009.

**Additional file 9. Table S7.** Top 20 downregulated KEGG and Reactome pathways for phospho-tyrosine or TiO2 enrichment in BLU9931-treated HCI-009.

## Data Availability

All data generated or analyzed during this study are included in this published article and its supplementary information files. The original data supporting these findings are available at any time upon request to the corresponding author.

## Abbreviations

DDA: Data-dependent acquisition
ER: Estrogen receptor
FDR: False discovery rate
FGFR: Fibroblast growth factor receptor
HCD: Higher-energy collisional dissociation
HER2: Human epidermal growth factor receptor 2
HRM-DIA: Hyper-reaction monitoring data-independent acquisition
iRT: Indexed retention time
LC–MS/MS: Liquid chromatography–tandem mass spectrometry
MS: Mass spectrometry
PDO: Patient-derived organoid
PDX: Patient-derived xenograft
PR: Progesterone receptor
RTK: Receptor tyrosine kinase
TNBC: Triple-negative breast cancer
