# Supplementary figures and images for: Evaluation of FGFR targeting in breast cancer through interrogation of patient-derived models
Source: Breast Cancer Res. 2021 Aug 3;23:82. doi: 10.1186/s13058-021-01461-4 (PMC8336364; doi:10.1186/s13058-021-01461-4)

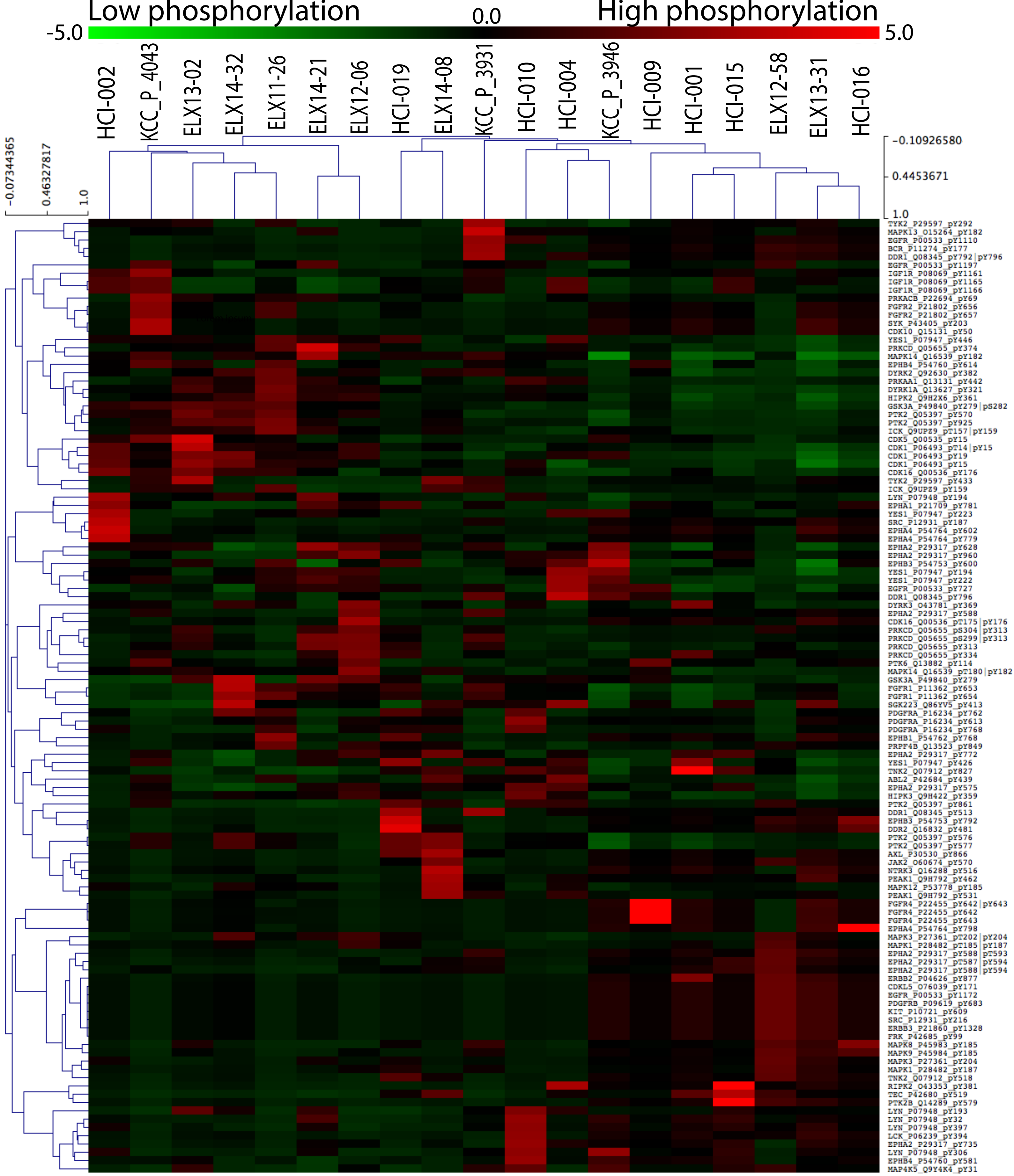

Supplement: Supplementary file 1 — Additional file 1. Fig. S1. High-resolution version of Figure 1a with individual kinases labeled on heat map. [file 13058_2021_1461_MOESM1_ESM.pdf]

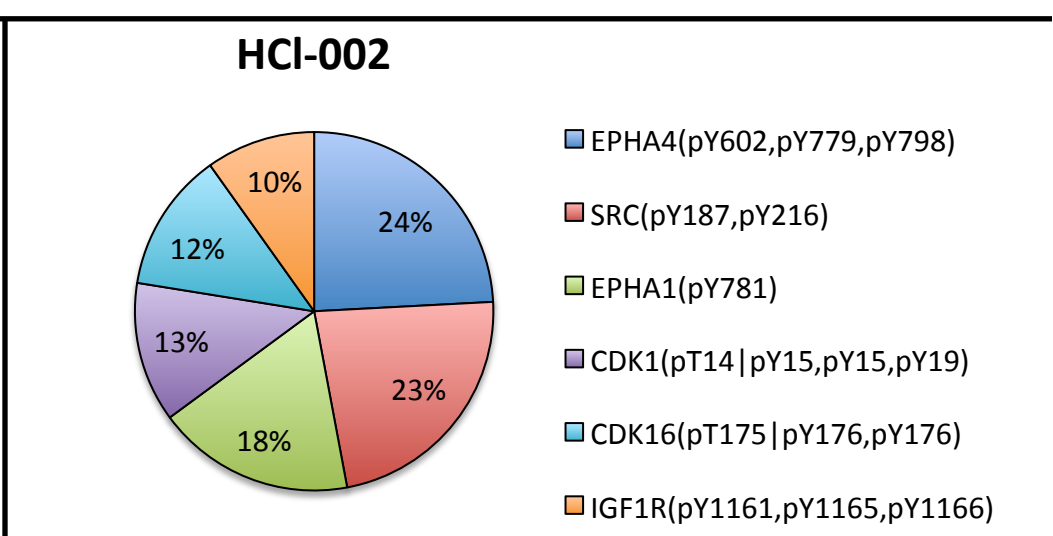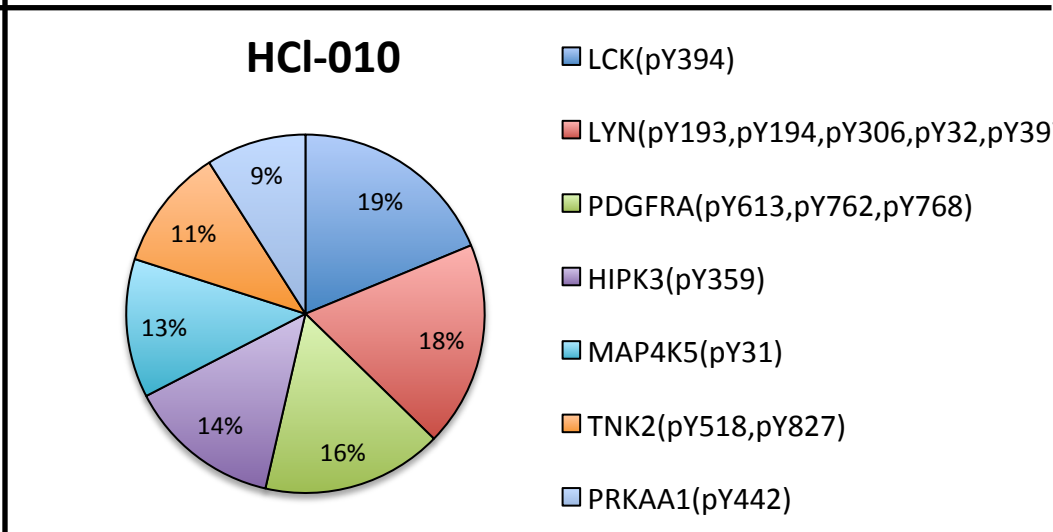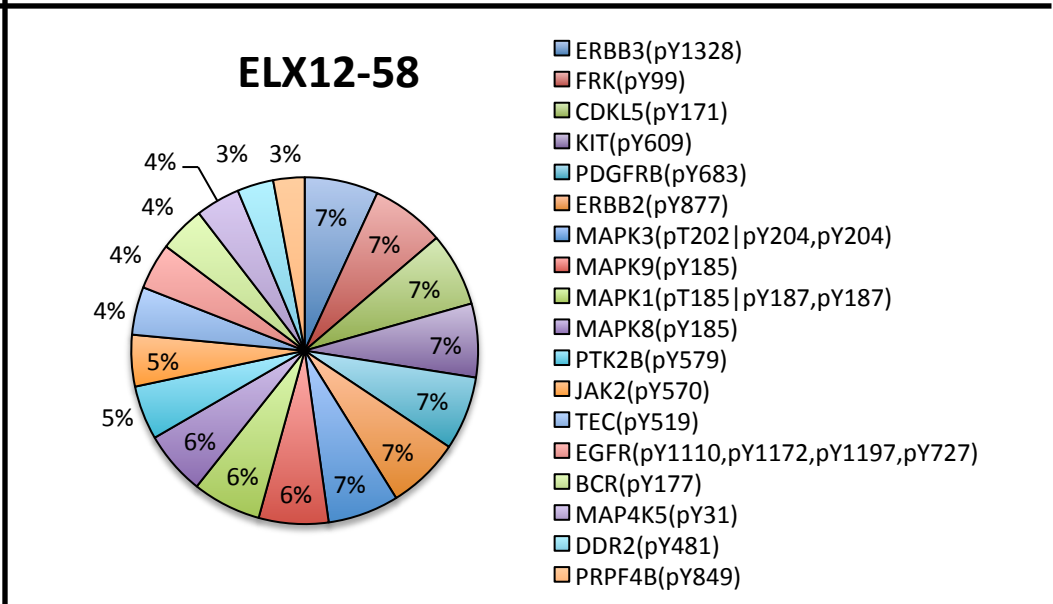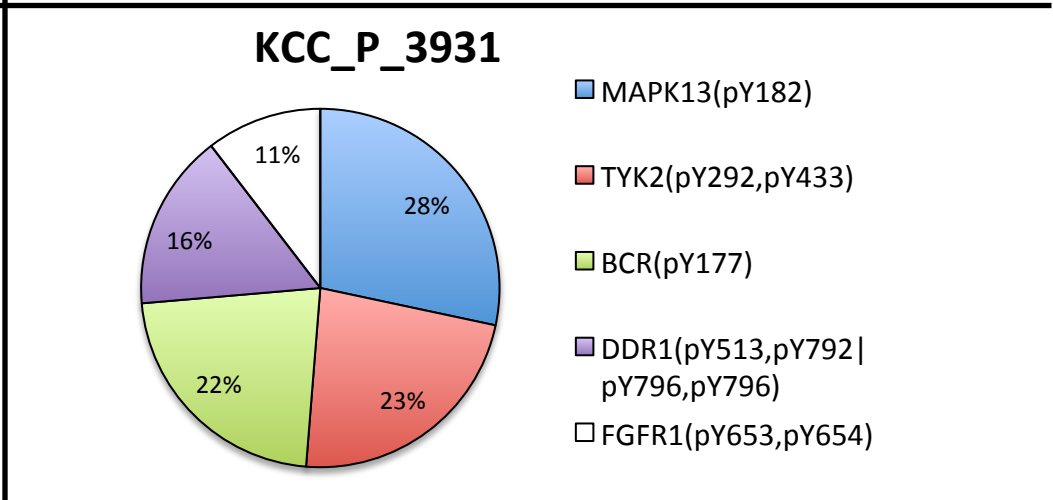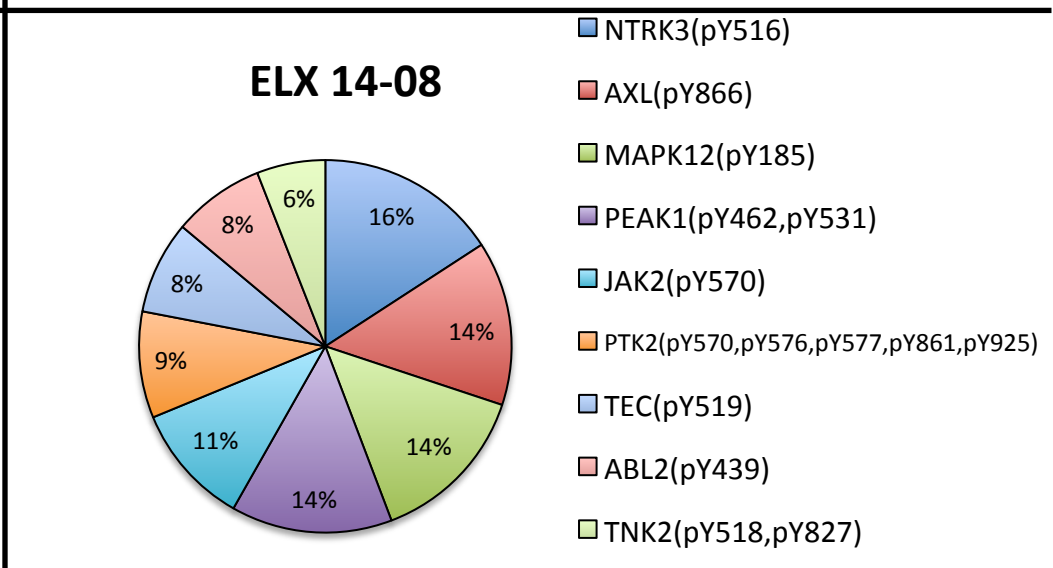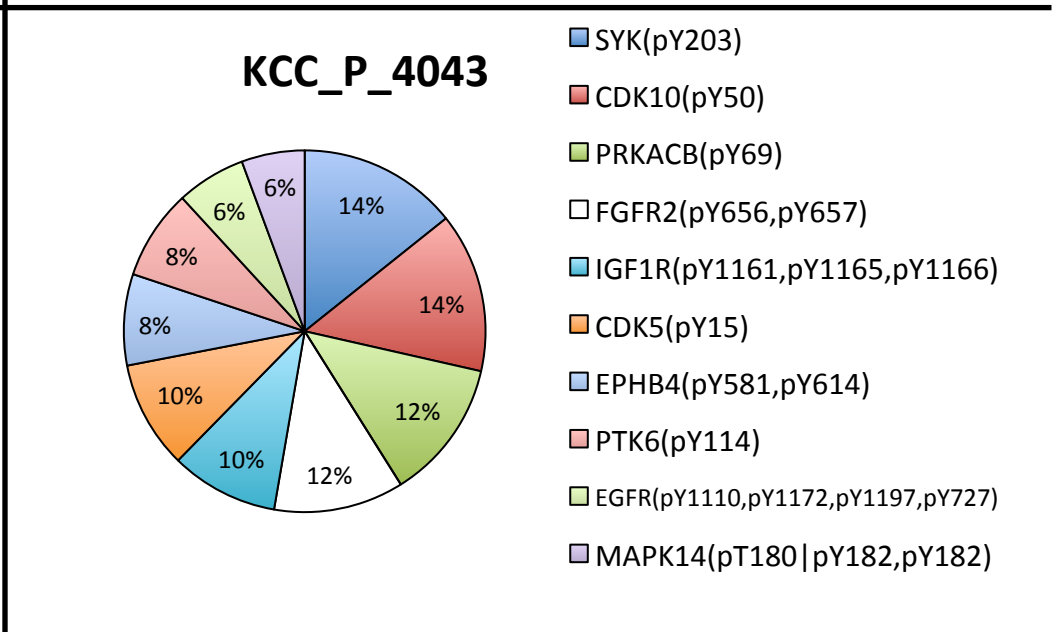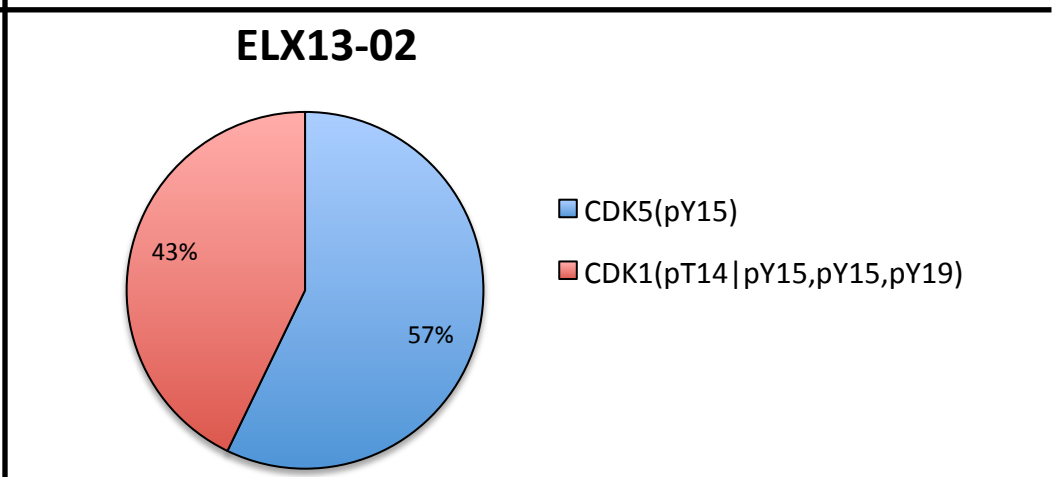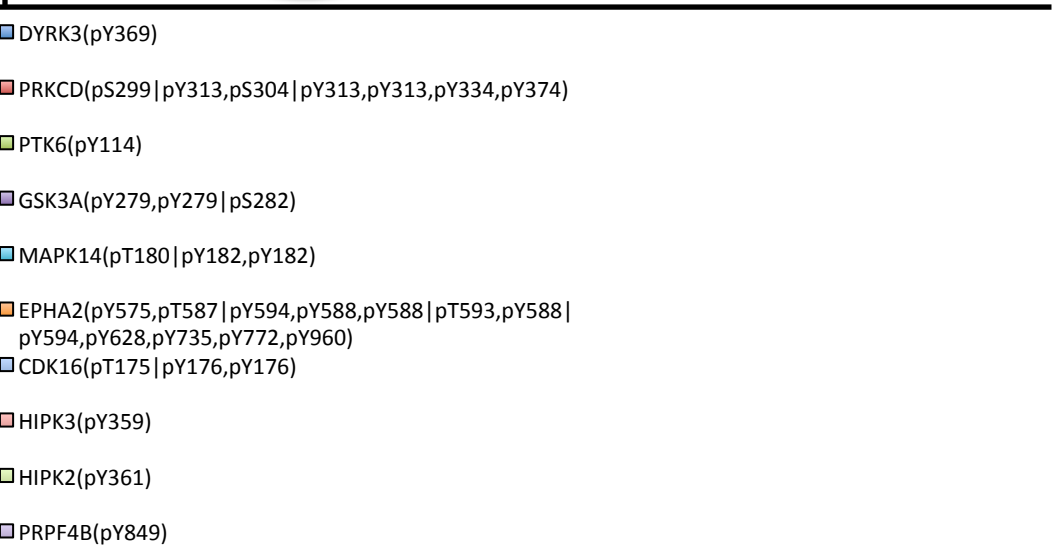

Supplement: Supplementary file 3 — Additional file 3. Fig. S2. Outlier kinases based on z-score of summed pY peptides of the kinase across PDX samples. Kinases with a z-score >1.5 were identified for each PDX sample and then expressed as a % of the summed Z scores. [file 13058_2021_1461_MOESM3_ESM.pdf]
