# Supplementary material for: Evaluation of FGFR targeting in breast cancer through interrogation of patient-derived models
Source: Breast Cancer Res. 2021 Aug 3;23:82. doi: 10.1186/s13058-021-01461-4 (PMC8336364; doi:10.1186/s13058-021-01461-4)

Supplementary Figure 3

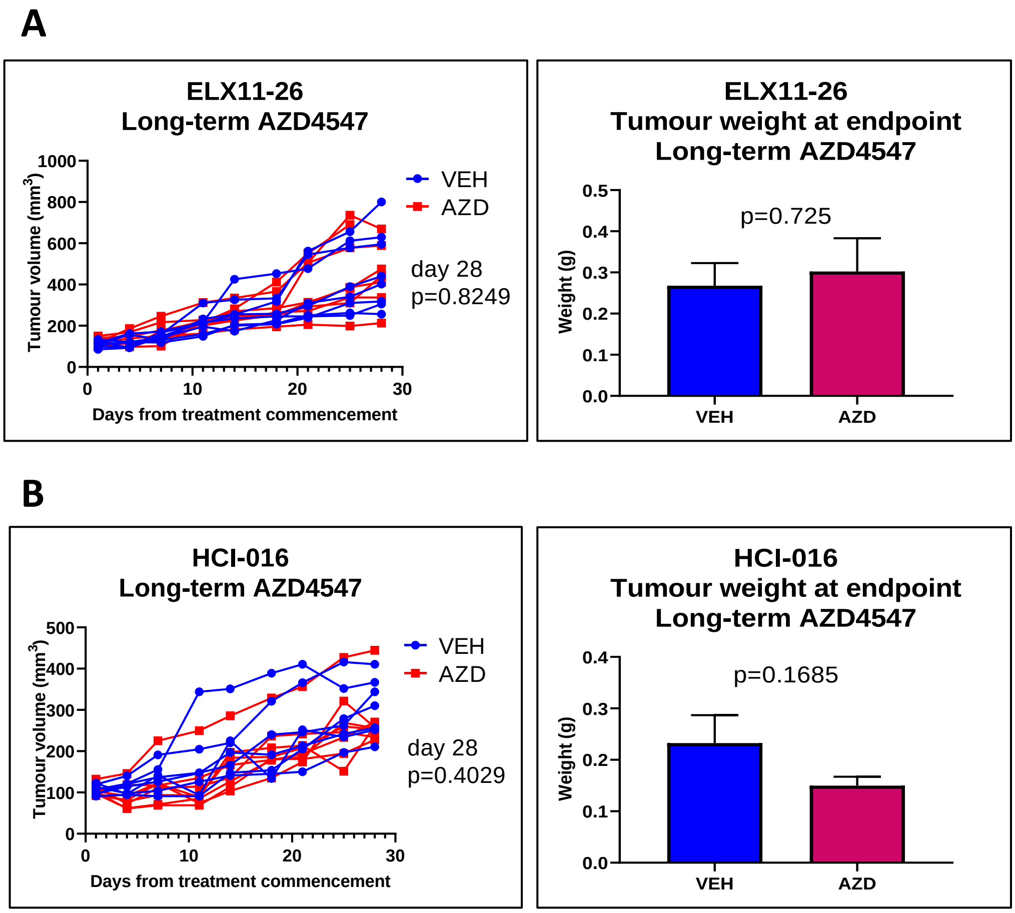

Supplementary Figure 4

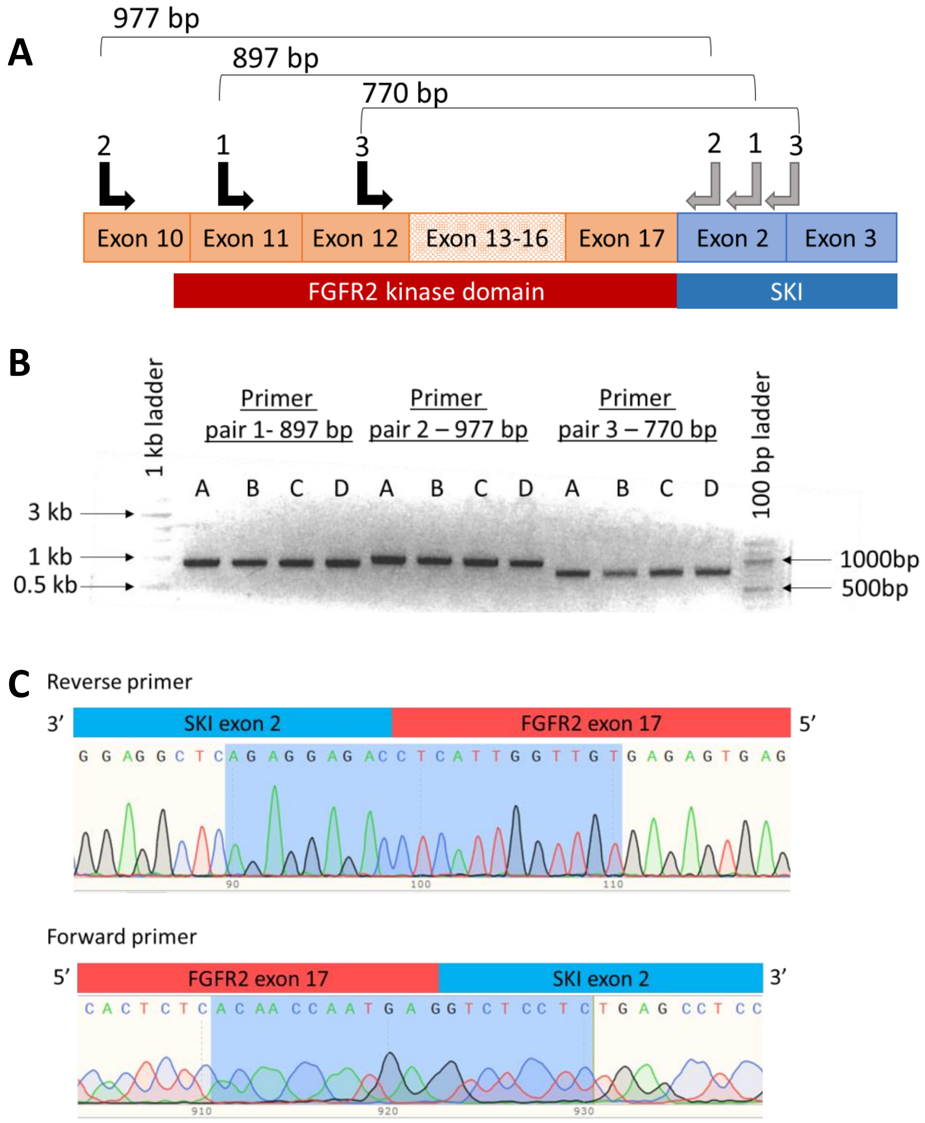

D

MVSWGRFICLVVVTMATLSLARPSFSLVEDTTLEPEDAISSGDDEDDTDGAEDFVSENSNNKRAP
YWTNTEKMEKRLHAVPAANTVKFRCPAGGNPMPTMRWLKNGKEFKQEHRIGGYKVRNQHWS
LIMESVVPSDKGNYTCVVENEYGSINHTYHLDVVERSPHRPILQAGLPANASTVVGGDVEFVCKV
YSDAQPHIQWIKHVEKNGSKYGPDGLPYLKVLKAAGVNTTDKEIEVLYIRNVTFEDAGEYTCLAG
NSIGISFHSAWLTVLPAPGREKEITASPDYLEIAIYCIGVFLIACMVVTVILCRMKNTTKKPDFSSQPA
VHKLTKRIPLRRQVTVSAESSSSMNSNTPLVRITTRLSSTADTPMLAGVSEYELPEDPKWEFPRDKL
TLGKPLGEGCFGQVVMAEAVGIDKDKPKEAVTVAVKMLKDDATEKDLSDLVSEMEMMKMIGK
HKNIINLLGACTQDGPLYVIVEYASKGNLREYLRARRPPGMEYSYDINRVPEEQMTFKDLVSCTYQ
LARGMEYLASQKCIHRDLAARNVLVTENNVMKIADFGLARDINNIDYYKKTTNGRLPVKWMAPE
ALFDRVYTHQSDVWSFGVLMWEIFTLGGSPYPGIPVEELFKLLKEGHRMDKPANCTNELYMMM
RDCWHAVPSQRPTFKQLVEDLDRILTLTTNE(EY)VSSEPPASIRPKTDDTSSQSPAPSEKDKPSSWL
RTLAGSSNKSLGCVHPRQRLSAFRPWSPAVSASEKELSPHLPALIRDSFYSYKSFETAVAPNVALAPP
AQQKVVSSPPCAAAVSRAPEPLATCTQPRKRKLTVDTPGAPETLAPVAAPEEDKDSEAEVEVESRE
EFTSSLSSLSSPSFTSSSSAKDLGSPGARALPSAVPDAAAPADAPSGLEAELEHLRQALEGGLDTKE
AKEKFLHEVVKMRVKQEEKLSAALQAKRSLHQELEFLRVAKKEKLREATEAKRNLRKEIERLRAENE
KKMKEANESRLRLKRELEQARQARVCDKGCEAGRLRAKYSAQIEDLQVKLQHAEADREQLRADL
LREREAREHLEKVVKELQEQLWPRARPEAAGSEGAAELEP

Supplementary Figure 5

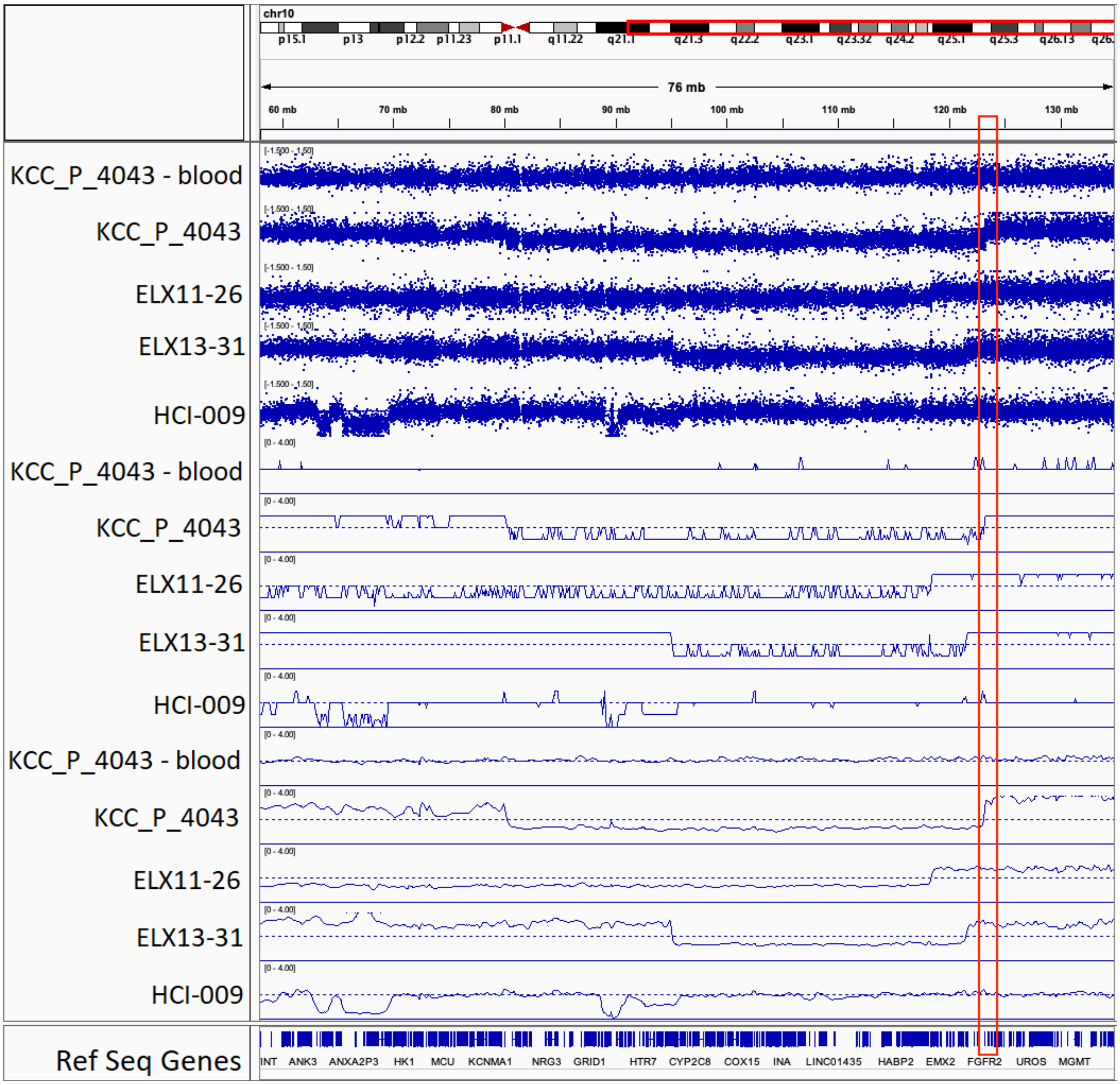

Supplementary Figure 6

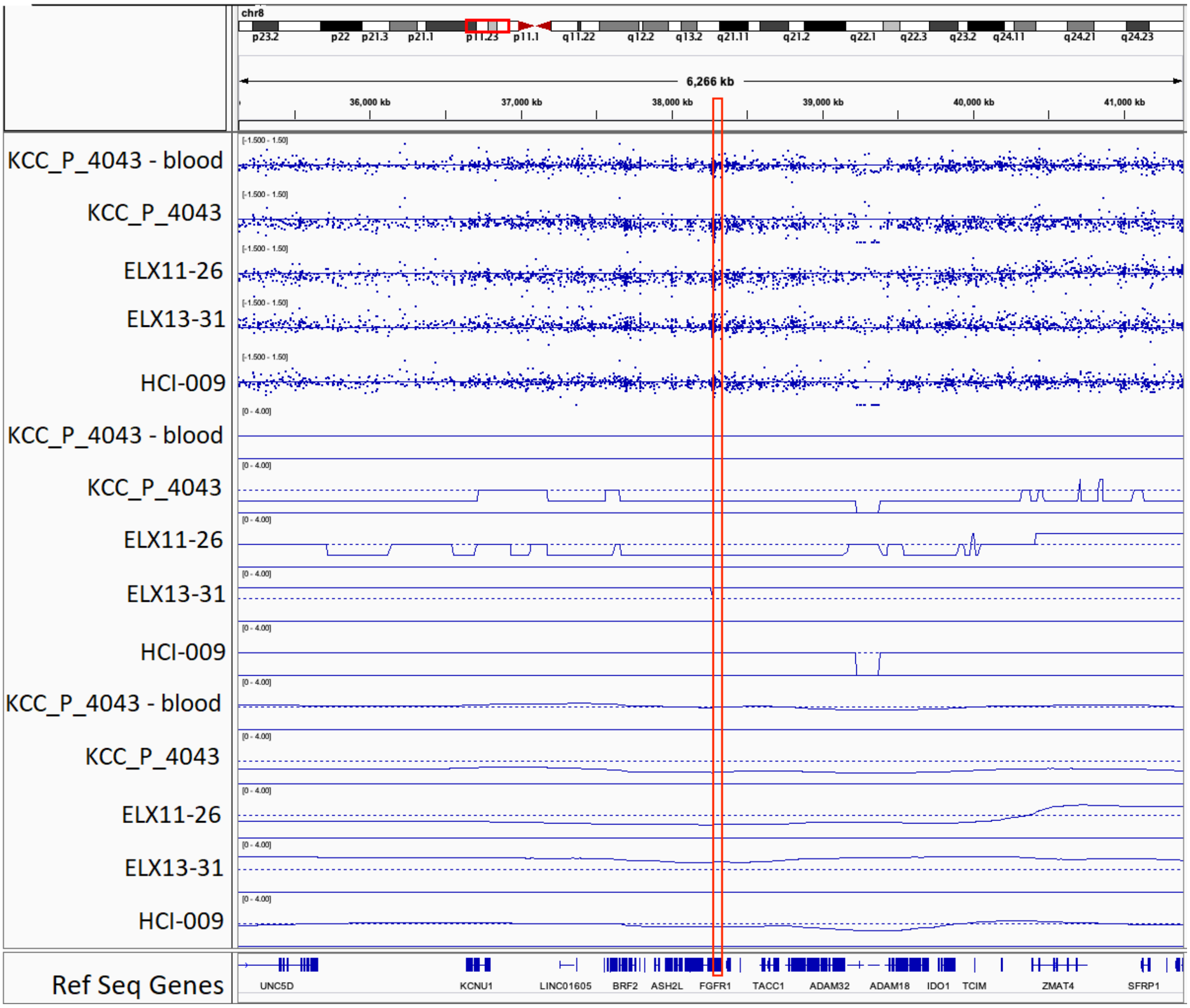

Supplementary Figure 7

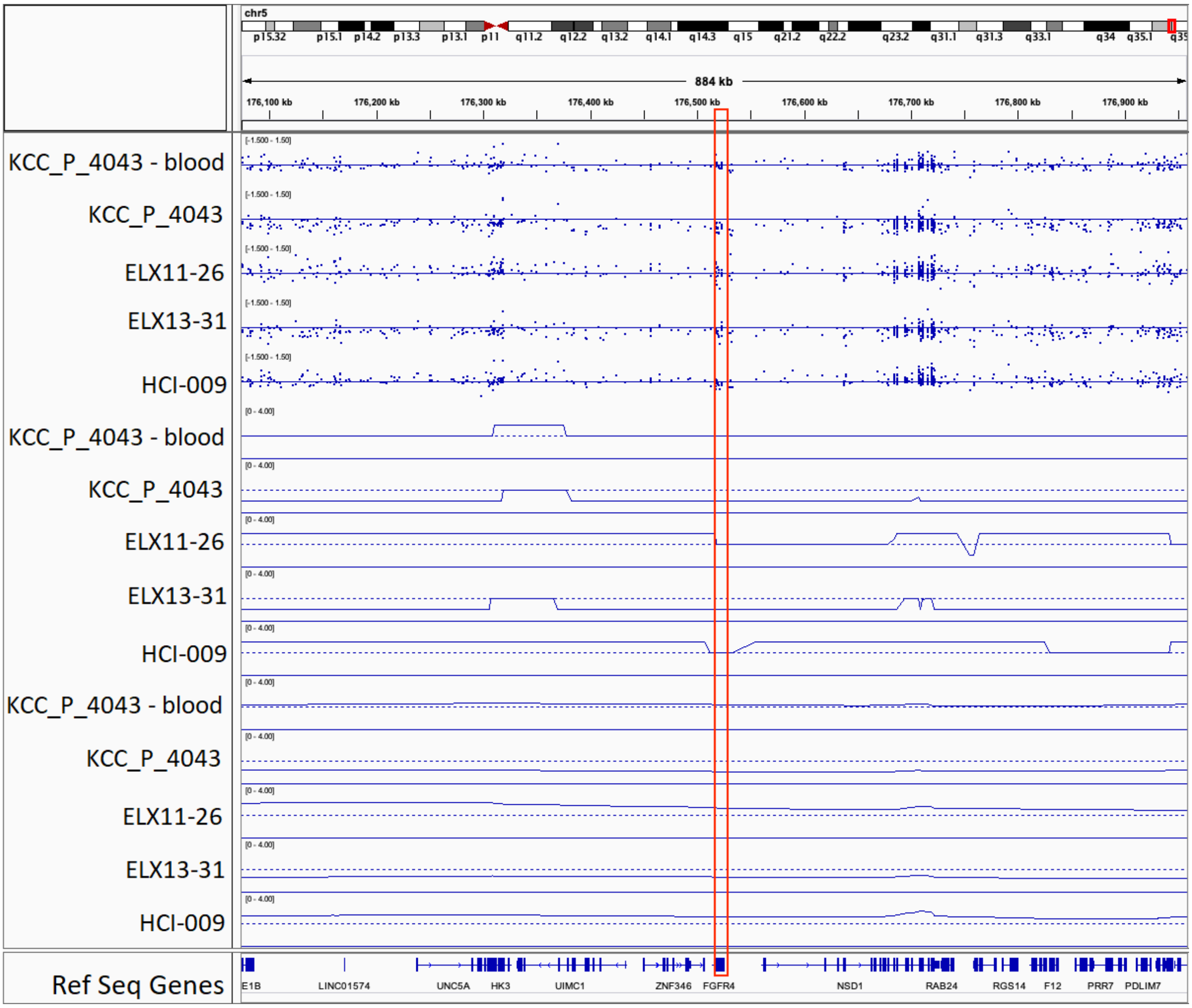

Supplementary Figure 8

A

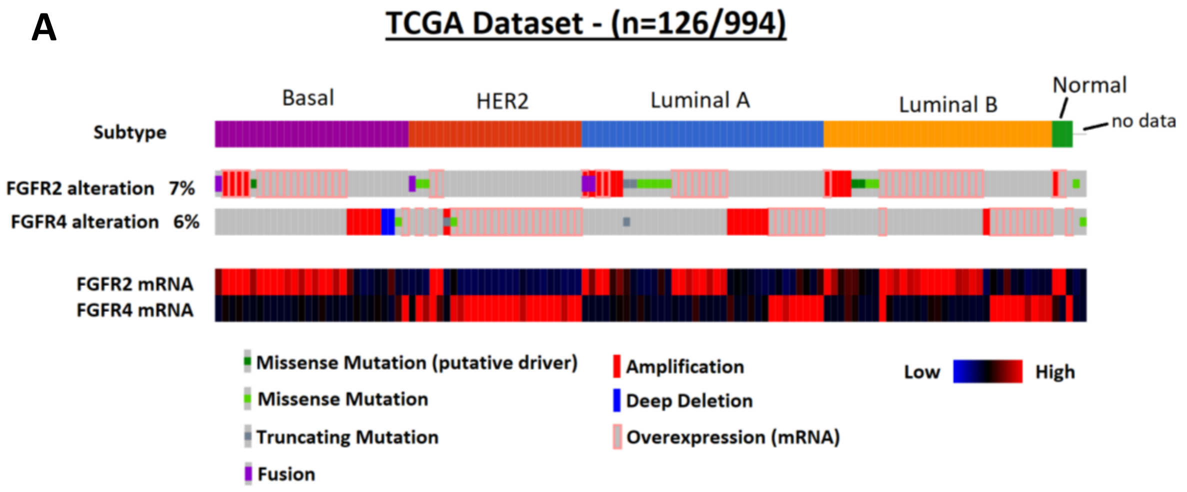

B

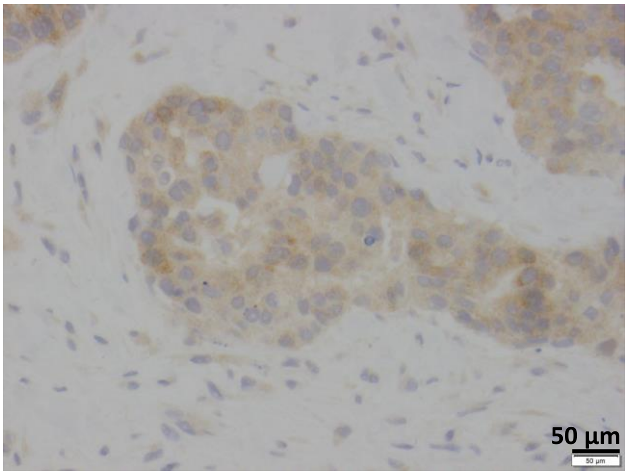

C

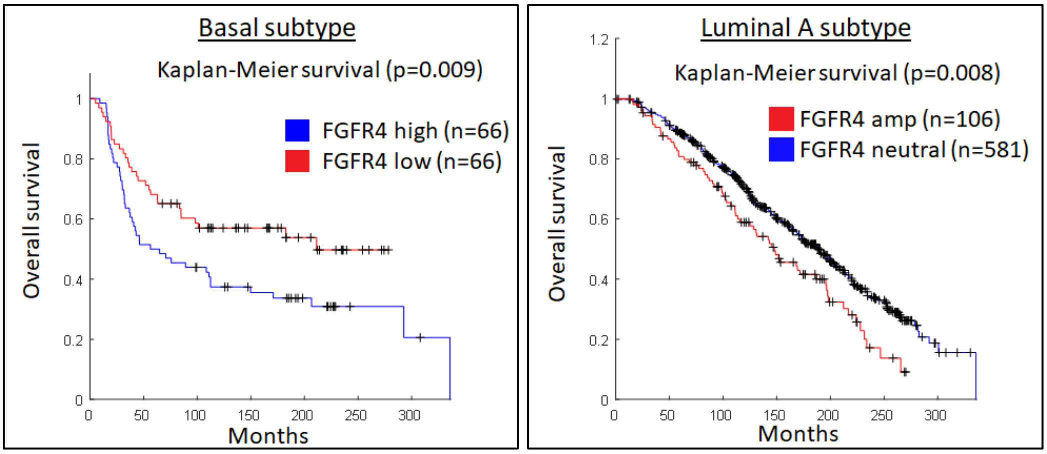

Supplement: Supplementary file 4 — Additional file 4. Fig. S3. Effect of FGFR1-3 inhibitor AZD4547 on a ELX11-26 and b HCI-016 PDX models. Mice were treated with vehicle control or AZD4547 for long term (28 d; 8 mice per group) and the tumor volume measured daily. Statistical significance was determined using an unpaired t test at the final timepoint (ELX11-26, p value = 0.825; HCI-016, p value = 0.403). Tumor weight at endpoint for the long-term treatment group was also measured and statistical significance was determined using an unpaired t test. Fig. S4. Identification of FGFR2-SKI fusion in KCC_P_4043. a Schematic of the primer pairs 1–3 targeted to the start of the FGFR2 kinase domain at exon 10 to 12 and at SKI exon 2 to 3 for PCR. Black arrows are the forward primers, gray arrows are the reverse primers. The predicted PCR product sizes are indicated. b FGFR2-SKI RT-PCR. The PCR products using primer pairs 1–3 from (a) were resolved by DNA gel electrophoresis and the bands were imaged using a fluorescent illuminator. c Sequence of the FGFR2-SKI fusion junction in KCC_P_4043 derived from RT-PCR products. d Amino acid sequence of the FGFR2-SKI fusion showing that the majority of the FGFR2 kinase domain (highlighted in yellow) is present in the FGFR2-SKI fusion. Only 2 amino acids, glutamate and tyrosine are missing (highlighted in green) from the FGFR2 kinase domain of the FGFR2-SKI fusion. Red, FGFR2; blue, SKI. Fig. S5. SNP arrays showing FGFR2 alterations in specific PDX. Track order as for Fig. 3c. 13-31 is a PDX with FGFR2 copy number gain included as a positive control. Fig. S6. SNP arrays showing FGFR1 alterations in specific PDX. Fig. S7. SNP arrays showing FGFR4 alterations in specific PDX. Fig. S8. FGFR2 and FGFR4 alterations in human breast cancer. a Frequency of FGFR2 and FGFR4 alterations in different breast cancer subtypes. Data were extracted from the TCGA Pan-cancer Atlas dataset in cBioPortal. Only patients with FGFR alterations are displayed for brevity. b FGFR4 positive immunoh [file 13058_2021_1461_MOESM4_ESM.pdf]
